# Multidimensional quantitative phenotypic and molecular analysis reveals neomorphic behaviors of p53 missense mutants

**DOI:** 10.1038/s41523-023-00582-7

**Published:** 2023-09-29

**Authors:** Anasuya Pal, Laura Gonzalez-Malerva, Seron Eaton, Chenxi Xu, Yining Zhang, Dustin Grief, Lydia Sakala, Lilian Nwekwo, Jia Zeng, Grant Christensen, Chitrak Gupta, Ellen Streitwieser, Abhishek Singharoy, Jin G. Park, Joshua LaBaer

**Affiliations:** 1 The Biodesign Center for Personalized Diagnostics, Biodesign Institute, Arizona State University, Tempe, AZ 85287 USA; 2 The School of Molecular Sciences, Arizona State University, Tempe, AZ 85287 USA; 3 The School of Life Sciences, Arizona State University, Tempe, AZ 85287 USA; 4 The Biodesign Center for Structural Discovery, Biodesign Institute, Arizona State University, Tempe, AZ 85287 USA

**Keywords:** Breast cancer, Oncogenes, Mechanisms of disease, Tumour heterogeneity

## Abstract

Mutations in the *TP53* tumor suppressor gene occur in >80% of the triple-negative or basal-like breast cancer. To test whether neomorphic functions of specific *TP53* missense mutations contribute to phenotypic heterogeneity, we characterized phenotypes of non-transformed MCF10A-derived cell lines expressing the ten most common missense mutant p53 proteins and observed a wide spectrum of phenotypic changes in cell survival, resistance to apoptosis and anoikis, cell migration, invasion and 3D mammosphere architecture. The p53 mutants R248W, R273C, R248Q, and Y220C are the most aggressive while G245S and Y234C are the least, which correlates with survival rates of basal-like breast cancer patients. Interestingly, a crucial amino acid difference at one position—R273C vs. R273H—has drastic changes on cellular phenotype. RNA-Seq and ChIP-Seq analyses show distinct DNA binding properties of different p53 mutants, yielding heterogeneous transcriptomics profiles, and MD simulation provided structural basis of differential DNA binding of different p53 mutants. Integrative statistical and machine-learning-based pathway analysis on gene expression profiles with phenotype vectors across the mutant cell lines identifies quantitative association of multiple pathways including the Hippo/YAP/TAZ pathway with phenotypic aggressiveness. Further, comparative analyses of large transcriptomics datasets on breast cancer cell lines and tumors suggest that dysregulation of the Hippo/YAP/TAZ pathway plays a key role in driving the cellular phenotypes towards basal-like in the presence of more aggressive p53 mutants. Overall, our study describes distinct gain-of-function impacts on protein functions, transcriptional profiles, and cellular behaviors of different p53 missense mutants, which contribute to clinical phenotypic heterogeneity of triple-negative breast tumors.

## Introduction

The tumor suppressor *TP53* remains the most altered gene in human cancer, mutated in almost 50% of all cancers. Unlike the classical tumor suppressor genes that are typically altered by indels and truncation mutations, more than 75% of the clinical mutations in *TP53* are missense mutations^1,2^, characteristic of oncogenes. This has led to the suggestion that cancer-causing characteristics of mutant p53 rely on both loss-of-function (LOF) and neomorphic gain-of-function (GOF) activities^3–7^. However, how different missense mutant p53 proteins promote cancer remains unclear. One hypothesis holds that missense mutations in the DNA binding domain (DBD) of the p53 transcription factor alter the DNA binding properties, affecting the transcription, downstream pathways, and the resulting cellular phenotypes. This raises another critical question of how differently each of these *TP53* missense mutations behave.

The p53 protein controls multiple cellular programs that suppress cancer and is activated by various stimuli which then mediate tumor suppression by transient cell-cycle arrest, induction of senescence, apoptosis and DNA-repair, called the canonical p53 functions^7,8^ and also, regulate cell metabolism, stem cell maintenance, invasion and metastasis prevention, called the non-canonical functions^9^. As p53 is a multi-functional protein, it is likely that each missense mutation affects these tumor suppressor functions differently.

p53 is a homo-tetrameric transcription factor, and *TP53* missense mutations in cancer occur mostly in the DBD (Supplementary Fig. 1a). While residues like R248 and R273 contact the minor groove and the major groove of DNA, respectively, other mutations in residues not directly contacting the DNA cause misfolding of the DBD and structural defects^10,11^. Mutant p53 proteins with abrogated or altered DNA binding fail to regulate the canonical transcriptional targets of wild type (WT) protein, leading to LOF effects. The missense mutant p53 monomers can form hetero-tetramers with WT p53 and hinder sequence-specific DNA binding, exerting dominant-negative (DN) effects^12^ and reducing the ability of WT p53 to bind to the target genes in presence of mutant p53, as confirmed by chromatin immunoprecipitation (ChIP)^13^. In addition to the DN effect, diverse neomorphic GOF effects of different p53 missense mutant proteins are also described, such as, transgenic mice with null vs. missense *TP53* alleles show a different tumor spectrum. For example, heterozygous *TP53*^*R270H/+*^ (human R273H) and *TP53*^*R172H/+*^ (human R175H) mice formed allele-specific tumor spectra differing from *TP53*^*+/−*^ mice^14–16^. *TP53*^*R270H/+*^ mice had primarily invasive carcinomas with increased tumor burden, whereas metastatic osteosarcomas were frequent in *TP53*^*R172H/+*^ mice compared to the *TP53*^*+/−*^ mice^17^. The heterozygous *TP53* state, associated with stabilization of mutant p53 and nuclear accumulation, is observed in many mutant *TP53*-driven cancers as well as during the early stages of tumorigenesis^18,19^. This in turn may amplify the GOF properties of the missense mutant p53 and reveal neomorphic functions not observed for WT or null conditions^20–22^. In cultured cells, mutant p53 proteins promote genomic instability, cell proliferation, apoptosis resistance, chemoresistance, cell migration and invasion^23–26^. In vivo, mutant p53 knock-in mice with homozygous mutations of R248Q and G245S showed early onset of different tumor types in comparison to the *TP53* null mice^27^.

Breast cancer is heterogeneous, comprising different subtypes with characteristic molecular features, prognoses, and therapy responses, broadly classified into basal-like, HER2 overexpressing and luminal types^28^. The basal-like subtype is typically ER, PR and HER2 receptor-negative, hence, triple-negative breast cancer (TNBC) and displays high grade, mitotic, aggressive, metastatic behavior, relapse with distant metastasis, and consequently has shorter time to progression and worse disease-free survival with the fewest treatment options^29–31^. TNBC is highly heterogeneous and in turn consists of six more subtypes^32,33^ with prevalent *TP53* mutations and somatic mutations in other genes, variable between and within tumors^34^. Somatic mutations in *TP53* are known to occur early in breast cancer^35^ and Ductal Carcinoma In-Situ (DCIS), preceding invasion, and the mutation frequency increases with the grade of DCIS tumors. More than 100 different *TP53* missense mutations are found in 30% of all breast tumors^36^ and occurs in 88% of basal-like, 26% of luminal, and 50% of HER2+ subtypes^37^. *TP53* mutations are also more prevalent in recurring tumors compared to primary breast tumors (41% and 23%, respectively), indicating the importance of *TP53* mutations for breast tumor progression^38^. However, the clinical significance of different missense mutations in breast cancer progression remains unclear.

As an atypical tumor suppressor, key questions on *TP53* remain unanswered: do different missense mutations exert unique GOF activities that contribute to oncogenesis; and by what mechanisms? Although studies on a few p53 mutants show potential neomorphic GOF of the individual mutants^20,21^, systematic comparisons between many different *TP53* missense mutations have not been reported. Therefore, we generated a panel of ten MCF10A cell lines, each expressing a different missense mutant p53 protein, and investigated their cellular and molecular characteristics. Our model system, MCF10A, a spontaneously immortalized but non-transformed mammary epithelial cell line with WT *TP53* is negative for ERα, PR, and HER2^39^ resembling TNBC^40^. MCF10A recapitulates the formation of normal 3D mammary acini-like spheroids and has been utilized to study mammary gland development and effects of genetic alterations on mammary cell transformation^41^. We characterized quantitatively the effect of each missense mutant p53 protein on multiple cancer phenotypes and performed RNA-Seq and ChIP-Seq studies to understand the molecular mechanisms driving the phenotypic differences, with specific focus on the cell invasiveness, the early step of metastasis frequently observed in TNBC. To quantify the association of various biological pathways with cellular phenotypes across the mutant p53 cell line panel, we specifically developed customized pathway analysis methods. Our integrated analyses from RNA-Seq and ChIP-Seq not only highlight neomorphic activities related to different *TP53* missense mutations, but also explain their cause by the molecular dynamics (MD) simulation studies.

## Results

### Characterization and quantification of multiple cancer phenotypes of the panel of mutant p53 protein-expressing MCF10A cell lines

We selected to study the ten most prevalent *TP53* missense mutations found in breast cancer patients (Supplementary Fig. 1a), among which four mutations (R248Q, R248W, R273C, and R273H) occur at residues that contact DNA (DNA contact mutations, hereafter), and the remaining six mutations (G245S, H179R, R175H, Y163C, Y220C, and Y234C) affect the overall structure of the DBD (structural mutants, hereafter). In addition to the differences among the individual mutations, it was of interest to see whether there are generalities in how these two groups of mutations affect cellular phenotypes, gene expression, and DNA binding properties. For the study, MCF10A cell lines stably expressing missense mutant p53 proteins and endogenous WT *TP53*, were established. Except for p53 mutant Y234C, western blots showed elevated levels of all mutant p53 proteins (Supplementary Fig. 1b), thus likely exerting DN effects on the endogenous WT p53 proteins. The mutant protein levels were comparable to those of breast cancer cell lines harboring missense *TP53* mutations such as HCC70 (*TP53*^*R248Q*^), MDA-MB-468 (*TP53*^*R273H*^), MDA-MB-231(*TP53*^*R280K*^), AU565 (*TP53*^*R175H*^), and SK-BR-3 (*TP53* ^*R175H*^).

To investigate quantitatively how the missense mutant p53 proteins affect cellular phenotypes, we performed cell-based functional assays for measuring major cancer hallmarks: 1) cell viability in absence of growth factors; 2) resistance to apoptosis; 3) cell migration; 4) cell invasion; 5) resistance to anoikis; and 6) changes in mammosphere morphology and polarity. In addition to the parental MCF10A cells with WT *TP53* (WT cells, hereafter), we included a control MCF10A cell line overexpressing the WT p53 protein (WT^OE^) to normalize for the protein-overexpression effects, and the phenotypic values in mutant p53-expressing cells were normalized as log2-transformed fold changes over the WT^OE^ values (Supplementary Table 1). Another control cell line with knocked-down WT p53 (WT^KD^) using shRNA constructs was also employed to underscore LOF *vs*. GOF activities of the missense mutants. To confirm the knock-down, as endogenous p53 proteins were hardly visible under normal growing conditions, we induced and stabilized the p53 protein levels by treating with doxorubicin and performing western blots. Supplementary Fig. 1c shows a clear reduction of p53 proteins in WT^KD^ cells compared to the parental MCF10A (or WT) cells, whereas WT^OE^ cells expressed a very high level of V5-tagged p53 proteins.

### Growth factor (GF)-independent survival

**Fig. 1:**
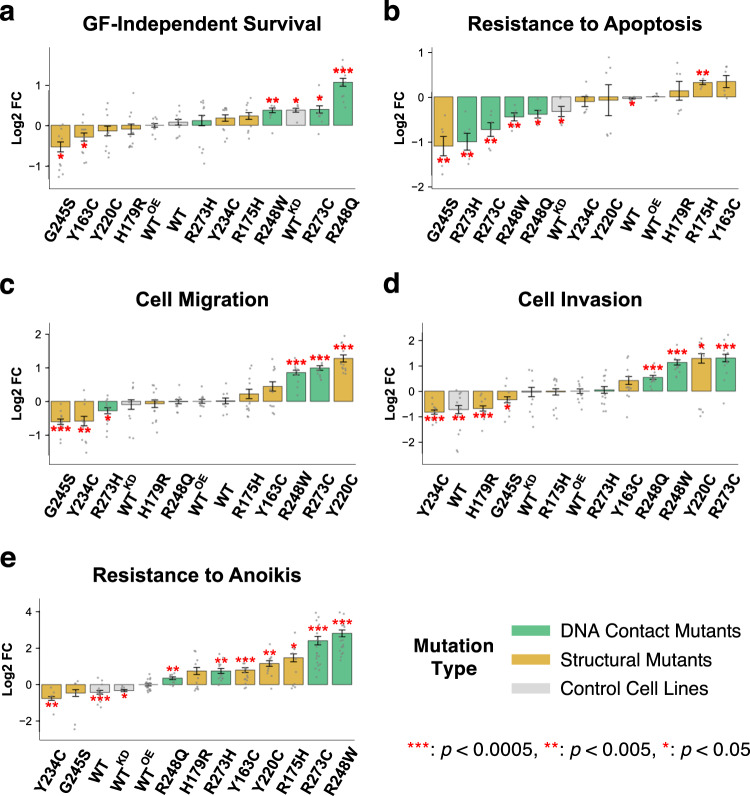
Effect of p53 missense mutations on hallmark phenotypes of cancer. Phenotypes were measured in three independent experiments with replicates, and the results were normalized to the log2 fold change over the phenotype of p53 WT^OE^ cells. The error bars represent the standard error of mean (SEM) values, and significant differences (two-sided single-sample *t*-test) from the WT^OE^ mean are indicated by red asterisks for indicated *p* values. In all plots, cells were sorted by each phenotype, from less (left) to more (right) aggressive. **a** Survivability of the cell line panel in absence of growth factors (serum and EGF) were measured by the CellTiter-glo assay. **b** Cell apoptosis was detected by the level of activity of caspase 3 and caspase 7, and the reciprocal of luminescence were used to measure the level of resistance to apoptosis. **c** Migration was assessed using the transwell assay. **d** Cell invasion was assessed using the transwell assay with Matrigel-coated chamber membranes. **e** To measure anoikis, the death index (ratio of dead cells and live cells) was calculated for each cell line, and the reciprocal values were used to measure the level of resistance to anoikis.

Cancer cells survive and sustain high cell proliferation rates even in the absence of growth factors. The cell line panel was incubated for 72 h in minimal medium with or without serum (S) and/or epidermal growth factor (EGF or E hereafter) in combinations (+S+E, +S−E, −S+E −S−E), and the viability was determined by the CellTiter-glo assay. Under normal culture condition (+S+E), no significant difference in cell viability was observed across the cell lines (Supplementary Fig. 2a). In the absence of serum and EGF (−S−E), WT^KD^ cells survived better than the WT or WT^OE^ cells (Fig. 1a). The R248Q mutant showed significantly greater viability than WT^KD^ cells, indicating neomorphic GOF activity over the LOF effects, and R273C, R248W, R175H, and R273H led to increased cell viability but to a lesser extent. The G245S cells did not survive without essential growth factors. In agreement with evidence that mammary epithelial cells require EGF for the initiation of survival signaling^42^, withdrawal of EGF (+S-E) decreased the viability of all other p53 mutant cell lines, except R248Q that also survived better than WT^OE^ cells without serum (Supplementary Fig. 2a). Under the GF-deprived condition (−S−E), enhanced proliferation was observed for the mutants R175H and R248Q compared to WT^OE^ cells (Supplementary Fig. 2b), demonstrating an increased capability of self-sustained proliferation. Likewise, mouse embryonic fibroblasts (MEFs) extracted from the p53^R172H/+^ (equivalent to human R175H) mice were more proliferative than p53^+/−^ MEFs^17^.

### Resistance to apoptosis

Triggered by p53 upon DNA damage, apoptosis is a barrier to cancer development, but mutations in *TP53* abolish apoptosis^43^ causing resistance to drugs such as doxorubicin. To examine doxorubicin-induced apoptosis, we measured the caspases 3/7 activities after 24-hour treatment with a range of concentrations (0.3, 0.7, 1.0, and 3.0 µM) of doxorubicin around the reported IC_50_ for breast cancer cells of 1.0 µM^44^. Nearly all cells died at 3.0 µM, and the results were excluded. Interestingly, when normalized to the WT^OE^ values, the trends of apoptosis levels across the cell lines were different for different doses of doxorubicin. As shown in Supplementary Fig. 3a, the trend at 0.3 µM was similar to that of 0.7 µM (Pearson’s *R* = 0.54) but not 1.0 µM (*R* = −0.40), and the response differentials between cell lines were much larger at 0.3 µM, indicating that different p53 mutations affect the sensitivity mostly at lower level of DNA damage. To summarize the relative apoptotic resistance across multiple doses of doxorubicin as a single representative value for each cell line, we employed a scoring method of comparing the area between the dose response curves of each cell line and the control WT^OE^ (Supplementary Fig. 3b). Overexpression of WT p53 did not affect significantly the sensitivity to apoptosis from the parental MCF10A cells (WT). The R175H cells were significantly more resistant than WT^OE^, whereas G245S, R273H, R273C, R248W, and R248Q were more sensitive (Fig. 1b). Interestingly, viability under GF deprivation did not always correlate with apoptosis resistance. Mutants like R248Q, R273C and R248W were more viable than WT^OE^ under GF deprivation but sensitive to cell apoptosis, whereas Y163C was less viable but more resistant to apoptosis.

### Cell migration and invasion

During cancer progression, adherent and polarized epithelial cells undergo epithelial-to-mesenchymal transition (EMT) that allows the cells to cut through the extracellular matrix in the basement membrane^45^. WT p53 suppresses EMT, whereas mutations in p53 proteins cause destabilization of cell-cell junctions, delocalization of β-catenin into cell cytoplasm^46^, and cell migration. When migratory potential of the p53 mutant cells was measured using the transwell plates (Fig. 1c and Supplementary Fig. 4a), Y220C, R273C, R248W, and Y163C cells were significantly more migratory than WT^OE^ cells, whereas the least migratory mutants were G245S, Y234C and R273H. Immunofluorescence staining for β-catenin and vimentin on the least (G245S), moderately (R175H), and most (Y220C and R273C) migratory cells showed that Y220C and R273C cells displayed typical mesenchymal characteristics including a spread “fan-like” morphology, delocalized β-catenin in the cytoplasm, and prominent vimentin (a mesenchymal cell marker) staining (Supplementary Fig. 4b).

Transwell chamber membranes coated with Matrigel were then used to measure cell invasion (Fig. 1d and Supplementary Fig. 5a). There was a clear distinction between weakly invasive Y234C, H179R, and G245S cells and more invasive R273C, Y220C, and R248W cells, which in general correlated with the levels of migration. Interestingly, the R175H cells showed a moderately increased migration but no change in invasiveness, demonstrating that promoting migration is not sufficient for making cells invasive. Further confirming this, when two of the migratory mutants R175H (moderately migratory but not invasive) and R248W (highly migratory and invasive) cells were seeded on a 3D matrix of the type I collagen-coated wells with a Matrigel overlay, R248W cells were detected up to 600 µm away from the base of the well, while very few R175H cells could invade the Matrigel overlay (Supplementary Fig. 5b). When compared to the metastatic MDA-MB-231 TNBC cells, the most migratory and invasive mutant p53-expressing cell lines, Y220C and R273C, were less migratory but equally invasive (Supplementary Fig. 6), putting these aggressive p53 mutations at an EMT induction level similar to fully developed metastatic cancer cells.

### Resistance to anoikis

To measure resistance to anoikis, which enhances tumor metastasis^47^ and is often caused by loss of functional p53^48^, the cell lines were grown in low-binding plates, mimicking the detachment from the basement membrane, for 7 days to form cell aggregates. The aggregates were stained with ethidium bromide (EtBr) and Vybrant violet to identify dead (i.e., EtBr-permeable) and live cells, respectively, (Supplementary Fig. 7) and the death index (the ratio of dead to live cell counts) was calculated (Fig. 1e). With higher death indices, Y234C and G245S cells had greater sensitivity to anoikis than WT^OE^, while the other mutant cells were more anoikis resistant, R273C and R248W being the most resistant and forming spherical aggregates of live cells (blue cells) detached from the 3D matrix (Supplementary Fig. 7). Notably, anoikis resistant R248W and R273C were more sensitive to apoptosis with doxorubicin treatment (Fig. 1b), indicating opposing effects of the mutations on DNA damage- vs. cell detachment-induced apoptotic programs.

### The 3D mammosphere morphology

The MCF10A cells form growth-arrested, polarized hollow spheres in 3D Matrigel matrix, called mammospheres, which resemble the glandular epithelial architecture of mammary acini^41^. It was reported that over-expression of WT p53 did not affect this phenotype, but cells without p53 (null or WT^KD^) and cells with a few hotspot *TP53* mutations led to depolarized, disorganized cell clusters with either filled or partially cleared lumen^49^. For high-throughput assessment of mammosphere formation, we developed a modified ‘on-top’ method in a 96-well format, where cells were seeded on top of a 3D Matrigel layer allowing mammosphere formation within a focal plane for automated confocal Z-stack imaging in a reduced time (~7–9 days).

**Fig. 2:**
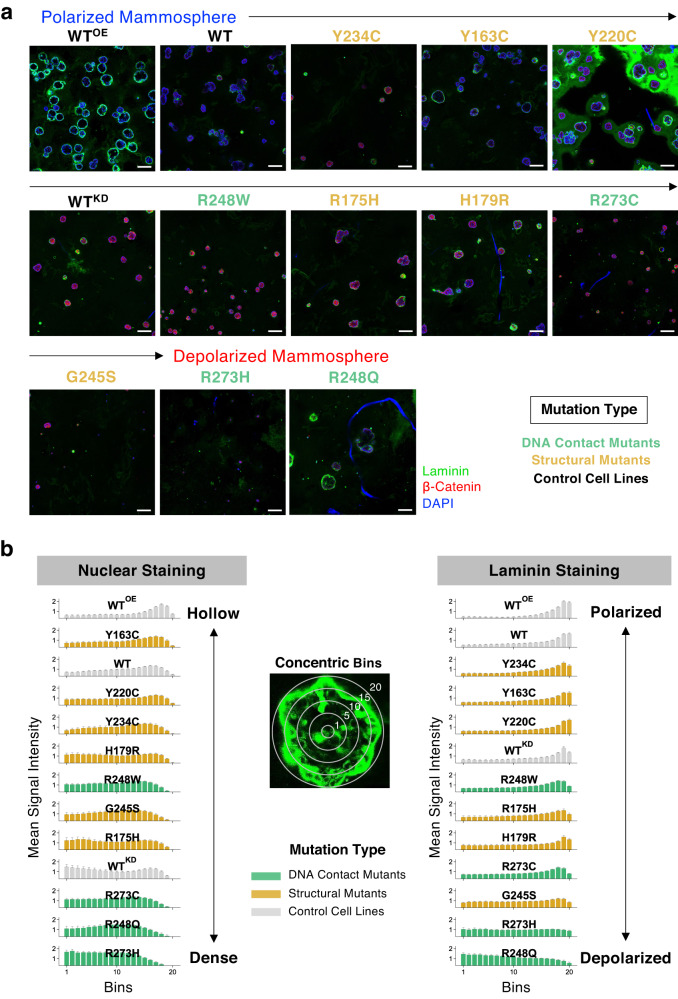
Morphology of the MCF10A p53 mutant-induced mammospheres. **a** Low-magnification image (scale bar: 100 μm) of the mammospheres formed by p53 missense mutants in Matrigel by on-top method stained for nucleus (DAPI, blue), laminin (green), and β-catenin (red). The cell lines were ordered by the degree of polarization shown below in (**b**) which shows the hollowness and laminin staining of the mammospheres. The nuclear DAPI intensity was measured in 20 concentric bins from the center of the mammospheres (left panel). The right panel shows the laminin staining intensity profiles across the bins, where laminin signal intensity in the inner bins indicate disruption of cell polarity. The intensity value data was normalized to the total number of pixels for each spheroid.

When the area of the equatorial cross-section of mammospheres derived from the cell lines (Fig. 2a) was measured, the WT and WT^KD^ cells formed mammospheres with comparable mean areas of 471 and 449 µm^2^, respectively, while the WT^OE^ cells formed largest hollow spheres with a mean area of 785 µm^2^ (Supplementary Fig. 8). All mutant p53-expressing cells formed smaller mammospheres with varying sizes, with the R273H cells forming the smallest spheroids with a mean area of just 65 µm^2^, and the maximum size variation was observed for R248Q, R248W, and G245S. Interestingly, mammospheres formed by the two most invasive mutants R273C and Y220C showed a significant size difference with mean areas of 198 µm^2^ and 475 µm^2^, respectively.

The hollowness of mammospheres was quantified by the area-normalized DAPI signal intensities in 20 consecutive concentric bins (or rings) from the center (Fig. 2b). The WT^OE^ cells formed mammospheres with cleared lumen like the WT cells, whereas WT^KD^ and all the p53 mutant cells except Y163C failed to clear cells at the center. The mutants R273H, R248Q, and R273C, which formed smaller spheroids, had prominent nuclear staining at the inner bins indicating filled lumens, whereas the remaining mutants had partially cleared lumens like the WT^KD^ cells.

An important morphological characteristic of normal mammospheres is the apical and basal polarization of the cells within the peripheral layer. When the area-normalized staining intensity of laminin, a protein that normally lines the outer basal side of the spheroids, was measured across the 20 concentric bins (Fig. 2b), WT^OE^ and WT cells had strong peripheral laminin staining, indicating normal epithelial cell polarity, whereas the laminin staining was detected inside the mammospheres for WT^KD^ cells, reflecting disrupted cell polarity (Supplementary Fig. 9). The R248Q cells showed the strongest laminin staining at the center, whereas the laminin intensity remained constant across the bins for the mutants R273H, G245S, and R273C that formed the dense mammospheres. Notably, G245S-derived mammospheres showed very low laminin levels overall, and the mammospheres formed by the least invasive and the most anoikis-sensitive Y234C cells had predominantly peripheral laminin staining.

### Different missense mutant p53 proteins induce heterogeneous neomorphic phenotypes

**Fig. 3:**
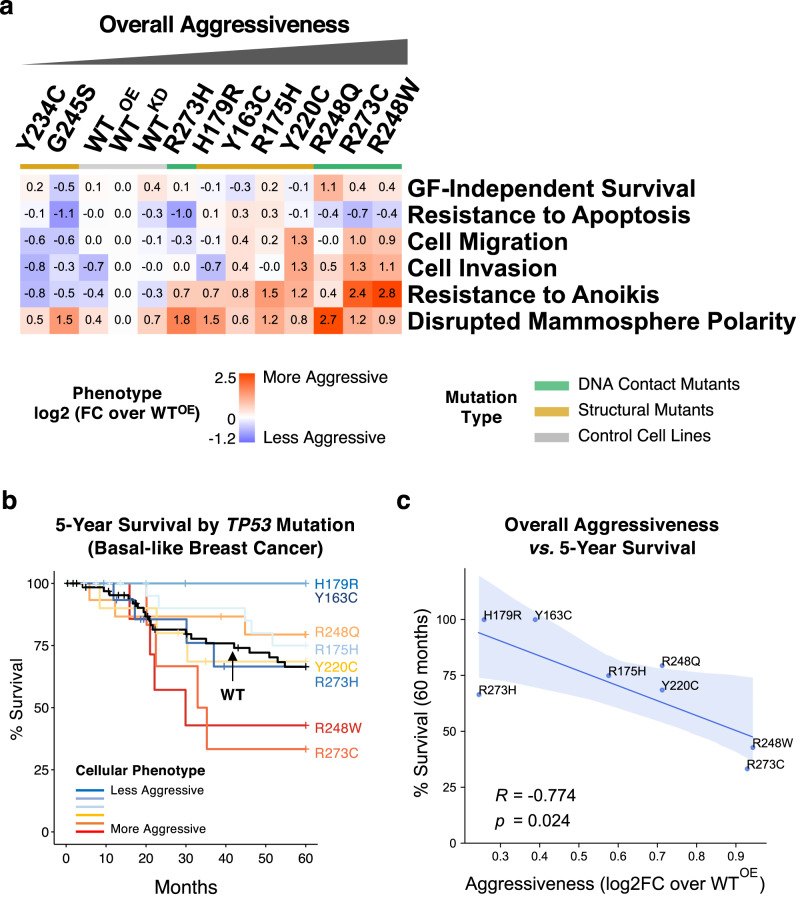
Cellular phenotypic heterogeneity of MCF10A cell lines expressing 10 different mutant p53 proteins. **a** The phenotype measurements for each of the p53 mutant-expressing cells were normalized (as log2-transformed mean fold changes) over those of the control p53 WT^OE^ cells and displayed as a heat map. The cell lines were sorted by the mean of the normalized values for all 6 phenotypes. The p53 mutants are color-coded by mutant type as indicated. The detailed results are in Supplementary Table 1**. b** Five-year survival rates of basal-like breast cancer patients with different missense *TP53* mutations were obtained from TCGA and METABRIC datasets and visualized by the Kaplan-Meier plot. Only the mutations with 3 or more corresponding samples were used. The mutations were color-coded by the phenotypic aggressiveness (red: more aggressive, blue: less aggressive), and the WT *TP53* curve is shown in black. **c** Correlation between the overall aggressiveness and the 5-year survival rates for different p53 mutations is shown with the Pearson’s correlation coefficient (*R*) and the *p* value. The blue bands represent the 95% confidence intervals.

As summarized in Fig. 3a and Supplementary Table 1, different missense p53 mutant proteins are functionally unequal, with distinct “neomorphic” phenotypes unlike those of the WT^KD^ cells, and reminiscent of the phenotypic heterogeneity observed in TNBC tumors^31,50^. Compared to the reference cell line WT^OE^, the ten p53 missense mutants organized into groups with less or more aggressive oncogenic behaviors, except mammosphere polarity, where all missense mutants led to partially or fully disrupted polarity. Across all phenotypes, p53 mutants R248W, R273C, and R248Q were the most aggressive, which showed increased migration/invasion, anoikis resistance and loss of cell polarity in mammospheres, whereas the Y234C and G245S mutants were the least aggressive. Since the expression levels of mutant p53 proteins varied (Supplementary Fig. 1b), we also examined whether the phenotypic differences were affected by the relative protein levels between the cells. However, we observed no significant correlation between protein levels and the phenotypic trends except for a moderate, but not significant, correlation to the *Resistance to Apoptosis* (Pearson’s *R* = 0.49, *p* = 0.224).

### Association of cellular phenotypes with overall survival of breast cancer patients with missense p53 mutations

To examine whether the overall and individual phenotypic trends were associated with clinical phenotypes, we have performed bioinformatics analyses on publicly available patient-based datasets of TCGA^51^ and METABRIC^52^. When the basal-like subtype of patients with either wild-type *TP53* (*n* = 67) or the ten missense *TP53* mutations (*n* = 90) used in our study were selected and grouped by the *TP53* mutation type, it was immediately noticeable that there were only two or fewer patients with either Y234C or G245S mutations, in agreement with their least aggressive behaviors in our phenotypic assays (Fig. 3a). When the 5-year overall survival rates of patients with the remaining 8 *TP53* mutations with 3 or more available samples were then compared along with the WT p53 group, there was a clear correlative trend between the overall phenotypic aggressiveness and the survival rate (Fig. 3b and Supplementary Fig. 10a). Patients with the most aggressive R248W and R273C mutations based on our cellular assay had the lowest survival rates, whereas those with less aggressive Y163C and R179R mutants showed higher survival rates with a marginal statistical significance (log-rank test *p* = 0.072) (Supplementary Fig. 10b). In addition, quantitative assessment confirmed a strong and significant inverse correlation (Pearson’s *R* = −0.774, *p* = 0.024) between the overall phenotypic aggressiveness and the survival rates (Fig. 3c). When the association of individual cellular phenotypes were tested, *Cell Invasion* and *Resistance to Anoikis* both showed significant inverse correlations with the survival rates (Supplementary Fig. 10c), indicating strong relevance of these cell-level phenotypes to the clinical representation of fully developed cancer. Despite the limited availability in clinical sample size, these suggest that the results obtained by using our cell-based model reflect reliably the clinical phenotypic aggressiveness and that the clinical phenotypes may be predisposed by the type of *TP53* missense mutations and the accompanying cellular changes acquired at the very early stage of cancer development.

### Transcriptomic profiling and integrative quantitative pathway analysis identified functional trends associated with phenotypes of mutant p53-expressing cells

Given that p53 is a transcription factor and that these missense mutations cluster in the DBD, we hypothesized that these diverse phenotypes might arise largely from disparate transcriptional regulatory functions of each mutant p53 protein with downstream changes in gene expression profiles and cellular pathways. Although non-transcriptional mechanisms, such as altered protein–protein interactions, might also contribute to the neomorphic phenotypic changes, in this study, we focused on the gene regulatory effects (RNA-Seq) and the DNA binding properties (ChIP-Seq) of different p53 mutant proteins, followed by comprehensive bioinformatics analyses to infer molecular mechanism underlying the phenotypic heterogeneity.

As we used unprovoked cells for phenotype measurements, we profiled the baseline transcriptome (i.e., without p53 inducers such as doxorubicin or irradiation) using RNA-Seq on the entire cell panel (Supplementary Table 2a). Principal Component Analysis (PCA) using the most variably expressed genes (*n* = 2383) across the cell lines positioned all p53 mutant-expressing cell lines separately from the control cell lines (Supplementary Fig. 11a), among which the R273H cell line was distantly positioned, indicating the most distinct gene expression profile. When overall aggressiveness was overlayed onto the PCA plot, the most aggressive R248W, R273C, R248Q, and Y220C mutants clustered in proximity (i.e., sharing similar gene expression profiles), while the less aggressive Y234C and G245S mutants spread out, indicating distinct gene expression profiles between them. No clear separation between different mutation types was observed (Supplementary Fig. 11b). When individual phenotypes were examined, more invasive, migratory, or anoikis-resistant p53 mutants shared relatively similar global gene expression profiles (Supplementary Fig. 11c).

To find biological pathways that were correlatively dysregulated with phenotypic trends among the 13 cell lines, we developed a set of three pathway analysis methods (Supplementary Fig. 12) tailored specifically for quantifying the associations between two sets of continuous variables (i.e., phenotypic scores vs. gene expression profiles across the cell lines), in contrast to conventional methods such as Fisher’s exact test for analyzing discretized data (e.g., non-invasive vs. invasive cells). We searched for pathways that were enriched among the genes with a strong expression-to-phenotype correlation across the 13 cell lines by using the Gene Set Enrichment Analysis (GSEA)^53^ (Supplementary Table 2a). We also computed the enrichment levels of pathways in each cell line by the single-sample GSEA (ssGSEA)^54^ and then measured correlation of the enrichment levels with the phenotypes. Lastly, we adopted a machine-learning approach of Partial Least Squares Regression (PLSR), a robust regression modeling method suitable for analyzing high dimensional data with small sample sizes^55,56^. Multivariate PLSR models were trained on expression values of a given pathway gene set to fit best to the phenotypic trends, and the pathway-to-phenotype association was assessed by measuring correlation between the observed and the model-predicted phenotypic values. For robust measurement of model performance, fivefold cross-validation was employed.

**Fig. 4:**
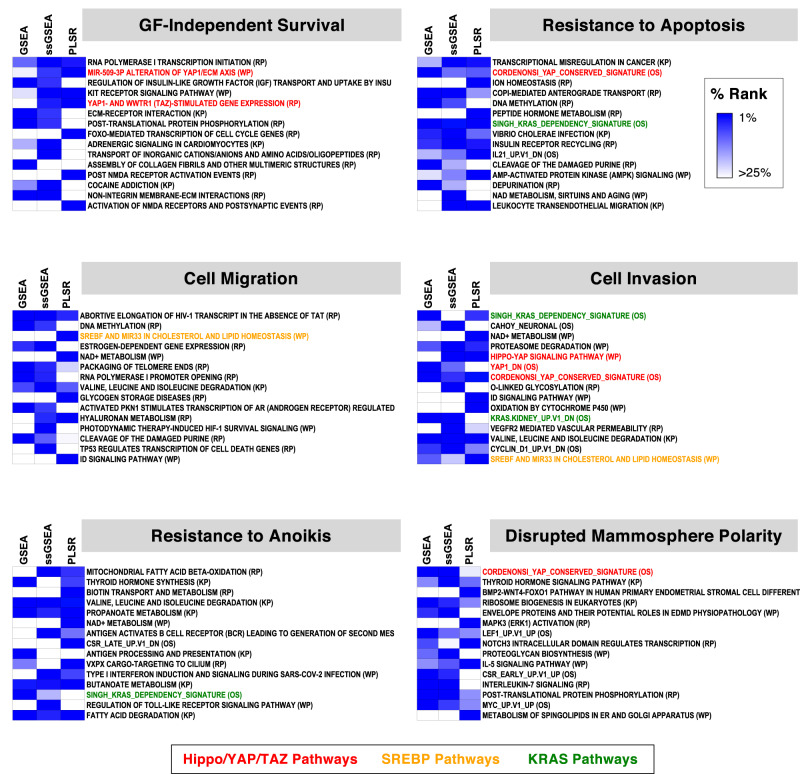
Transcriptomic profiles of mutant p53-expressing MCF10A cell lines and phenotype-associated pathways. For the top 15 phenotype-associated pathways for each phenotype selected based on the minimum rank in GSEA, ssGSEA, and PLSR results, the percentile ranks are displayed as heat maps. The pathways discussed in texts are color-coded, as indicated. KP KEGG pathways, RP Reactome pathways, WP WikiPathways, and OS MSigDB Oncogenic Signatures. RNA-Seq and pathway analysis results are in Supplementary Table 2.

A total of 2093 pathway terms were ranked for phenotype association by the enrichment *p* values (GSEA, Supplementary Table 2b), the absolute phenotype correlation (ssGSEA, Supplementary Table 2c), and the correlation between observed and model-predicted phenotypes (PLSR, Supplementary Table 2d**)**. When the top terms for each phenotype were selected by using the minimum percentile ranks of the three methods as the overall summary metric, several terms were represented broadly across different phenotypes, including multiple signaling and metabolic pathways (Fig. 4). Most notably, multiple Hippo/YAP/TAZ pathway-related terms were observed for cell invasion, GF-independent survival, resistance to apoptosis, and disrupted mammosphere polarity, such as “*Hippo-YAP Signaling Pathway*”, “*YAP1_DN*” (down-regulated genes upon overexpression of YAP1), and “*Cordenonsi YAP Conserved Signature*” (evolutionary conserved downstream target gene signature of YAP). Especially for invasion, the Hippo pathway was significantly enriched among the highly ranked gene sets (GSEA *p* = 0.0004). In comparison, several KRAS-related terms were also observed, but the enrichment was not significant (GSEA *p* = 0.64). We then quantified relative association strength of each pathway to phenotypes by performing PLSR on ssGSEA values of entire gene sets (e.g., WikiPathways) with cross-validated feature selection.

**Fig. 5:**
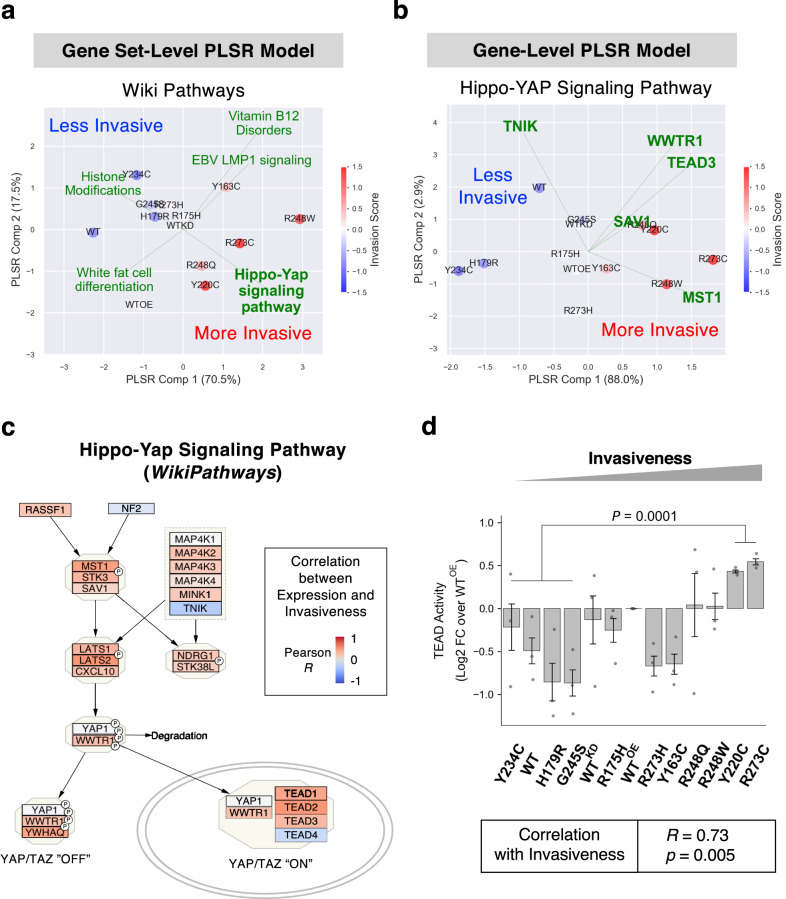
The Hippo/YAP/TAZ pathway is strongly associated with invasiveness of cells expressing different p53 mutants. **a** Biplots for a PLSR model trained on ssGSEA scores of the entire pathway terms in WikiPathways database and the vector of invasiveness is shown. The model was based on top 5 pathway terms that were identified by cross-validated forward feature selection. Cell lines (colored by invasiveness) were transformed and projected on a 2-component space, and the explained variance of invasiveness by each component is shown in the axis labels. The loadings of pathway terms were scaled to fit the data range and displayed as green lines. **b** Biplot for a PLSR model trained on expression values of genes in the *Hippo-YAP Signaling Pathway* (WikiPathways) and the vector of invasiveness is shown. Analysis performed as described for Fig. 5a. **c** In the WikiPathways diagrams for the Hippo/YAP/TAZ pathways, individual genes were color-coded by the Pearson correlations between gene expression levels and invasiveness across the 13 cell lines. **d** Transcriptional activity of TEAD proteins was measured in the 13-cell line panel in triplicates by a cell-based luciferase reporter assay. The luminescence values were normalized to the log2 fold changes over the WT^OE^ values. The difference between a group of two more invasive cells (R273C and Y220C) and four less invasive cells (Y234C, WT, H179R, and G245S) was tested by the two-sided Student’s *t*-test. Pearson’s correlation between the normalized TEAD activities and the invasiveness across the cell lines was also calculated (bottom box).

Figure 5a shows that the fitted PLSR model with top 5 invasion-associated pathways were able to explain 88% of the phenotypic variance and project them in 2D space by their invasiveness from the top left (less invasive) to the bottom right (more invasive). Further, the “*Hippo-YAP Signaling Pathway*” was identified as the top contributing pathway to increased invasiveness during the feature selection process. The Hippo/YAP/TAZ pathway regulates a wide range of cancer phenotypes including cell proliferation, survival, EMT, anchorage independent growth, cell migration, invasion, and stemness^57–59^. In addition, top-ranked included pathways related to SREBP (or SREBF) (Fig. 4), a master transcriptional regulator of mevalonate/cholesterol metabolism and a known upstream regulator of the Hippo pathway acting via Rho GTPases^60^. Figure 5b shows a 2D component projection (explaining >90% of phenotypic variance) of cell lines by a PLSR model trained with the top 5 feature-selected genes in the Hippo pathway, where expression levels of *MST1* and *TNIK* contributed positively or negatively, respectively, to invasiveness in the different mutant p53 background. When expression of the Hippo pathway genes was examined, most of the genes were highly expressed in invasive cells, with *MST1* (or *STK4*), *LATS2*, and *TEAD2* strongly correlating with invasiveness (Fig. 5c and Supplementary Fig. 13a). In addition, expression of a large fraction of the downstream target genes of YAP/TAZ activation in the *Cordenonsi YAP Conserved Signature* also positively correlated with invasiveness (Supplementary Fig. 13b). These suggest that coordinated transcriptional regulation of the Hippo pathway genes downstream of different mutant p53 proteins may govern invasiveness.

### Dysregulation of hippo pathway correlated with invasiveness of cells with different p53 mutants

We then sought functional confirmation if the Hippo pathway activity was concordantly regulated with invasiveness of the p53 mutant cell lines, which was significantly associated with survival rates of basal-like breast cancer patients (Supplementary Fig. 10c). As depicted in Fig. 5c, the tumor suppressor activity of the Hippo pathway leads to phosphorylation of the YAP/TAZ proteins by LATS1/2 kinases, causing their cytoplasmic retention and degradation. In contrast, the unphosphorylated forms of YAP/TAZ drive oncogenesis by partnering with TEAD transcription factors to increase transcription of the relevant targets^61^. To test whether activity of the Hippo pathway correlated with cell invasion, we measured the transcriptional activity of TEAD protein (i.e., the functional output of YAP/TAZ activation) in cells by transfecting with the luciferase reporter plasmid with TEAD-binding sequence motifs. As shown in Fig. 5d, the TEAD-mediated transcriptional activation was significantly correlated with invasiveness (Pearson’s *R* = 0.73, *p* = 0.005) across the 13 cell lines. Further, the two most invasive cell lines, R273C and Y220C showed significantly higher (two-sided *t*-test *p* = 0.0001) TEAD activities than those of the four least invasive cells. These demonstrated that the Hippo/YAP/TAZ/TEAD axis may be a key contributor for phenotypic heterogeneity determining the invasiveness of the cells expressing different missense p53 mutations, in agreement with the reported role of TAZ in breast cancer development and aggressive phenotypes in cell/animal models^62^ and breast carcinomas^63^. In our RNA-Seq data, expression of *WWTR1* (the gene symbol of TAZ*)* but not *YAP1* was also elevated in invasive cell lines (Fig. 5c and Supplementary Fig. 13a), implying the selective regulation of *TAZ* transcription by different mutant p53 proteins in promoting cell invasion.

### Association of the Hippo pathway dysregulation with basal-like molecular subtype of breast tumors and survival rates

To investigate whether the dysregulated Hippo/YAP/TAZ pathway signature identified from the MCF10A model is relevant for other cancer cell lines and tumors, we performed bioinformatics analysis on the Hippo pathway activities in a broad spectrum of breast cancer cell lines in CCLE^64^ as well as patient tumor sample data in TCGA and METABRIC databases. ssGSEA analysis on RNA-Seq data showed that elevation of genes in multiple Hippo-related pathway was a distinct signature of the basal-like breast cancer subtype among cell lines (Supplementary Fig. 14a, left panel) as well as tumors (Supplementary Fig. 14b, c, left panels). Interestingly, when analyzed by PCA based on the pathway enrichment scores, the Hippo pathway signature was more strongly associated with the basal-like subtypes of cell lines and tumors than other major cancer-associated pathways including p53, PI-3K, or Ras pathways (Supplementary Fig. 14, right panels), as indicated by the higher Calinski-Harabasz (CH) Indices of the Hippo pathway, which measured the strength of clustering of the basal-like sample group relative to other subtypes. Therefore, more aggressive p53 mutants may push the cell phenotypes towards more basal-like by inducing selectively ectopic activation of the Hippo pathway.

We then examined whether the elevated Hippo/YAP/TAZ pathway signature was associated with clinical phenotypic aggressiveness. When the basal-like samples in TCGA and METABRIC were selected and clustered by ssGSEA enrichment scores for the Hippo-related pathways, three clusters with high, mid, and low levels of enrichments were observed (Supplementary Fig. 15a). When we then compared the 5-year survival rates, the Hippo-high/mid groups had poor survival with a marginal statistical significance (log-rank test *p* = 0.08, Supplementary Fig. 15b, top panel). Importantly, when the same method was applied to other cancer-related pathways, we didn’t observe any difference between the sample groups with higher and lower pathway enrichments (Supplementary Fig. 15b, bottom panels). These suggest that dysregulation of the Hippo/YAP/TAZ pathway is a district signature of clinically aggressive tumors within the basal-like subtype.

### ChIP-Seq uncovered heterogeneity in DNA binding capacity and preference

The p53 protein is a transcription factor with approximately 300 known target genes, which binds to a well-conserved motif^65^. To test how different missense mutations affect the DNA binding properties, particularly regarding invasiveness, we performed ChIP-Seq on two invasive R273C and Y220C and two less invasive R273H and Y234C cell lines by using an antibody against the V5 tag fused at the C-term of p53 mutant proteins. We also included the WT^OE^ cells as a positive control and the WT cells expressing only endogenous WT p53 as a negative control. To enable an integrated analysis, we matched the RNA-Seq experimental conditions using cells under unstimulated culture conditions, which contrasts with other p53 ChIP-Seq experiments performed after treating cells with doxorubicin or 5-FU to elevate the p53 protein levels and induce DNA binding^66,67^. Thus, p53 DNA occupancy levels in our setting were expected to reflect the lower tonic conditions of the early-stage *TP53*-mutated tumor cells in the absence of excessive DNA damage or cellular stresses.

**Fig. 6:**
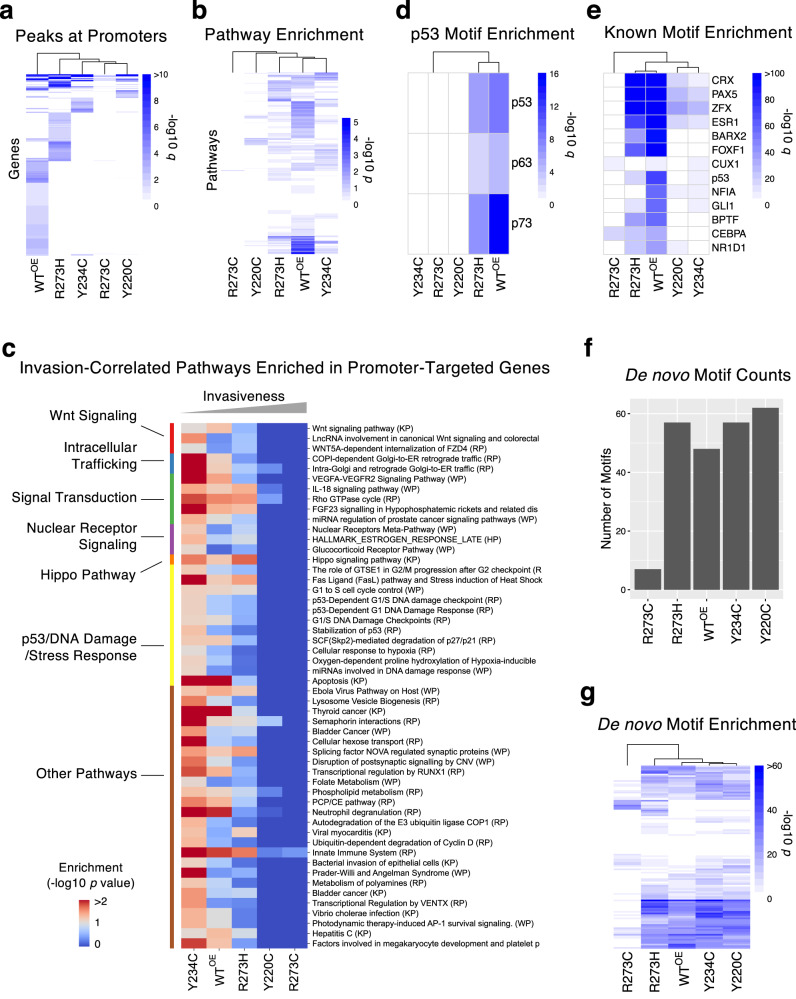
Characterization of DNA binding properties of 4 different p53 missense mutant proteins by ChIP-Seq. **a** Genes that were targeted (peak detection *q* < 0.05) by WT and mutant p53 proteins at the promoter region are shown as a heat map. The colors represent the −log10 *q* values. **b** Enriched pathways (hypergeometric test *p* < 0.05) in the identified targets of WT and mutant p53 proteins are shown as a heat map of −log10 *p* values. Representative results based on the WikiPathways pathway set are shown. **c** Enrichment of the canonical DNA binding motif of the p53 protein family in promoter regions for WT and mutant p53 proteins. The color represents −log10 (hypergeometric test *q*). **d** Invasion-correlated (Pearson’s correlation *p* value < 0.05) pathways that were enriched in target genes (hypergeometric test *p* value < 0.1) of WT or mutant p53 proteins. The terms were sorted by the pathway groups (shown in left). **e** Enrichment of top known transcription factor binding motifs within identified peaks for WT and mutant p53 proteins. The color represents −log10 (hypergeometric test *q*). **f** The bar plot shows the number of de novo motifs (*p* < 1.0e-10) found for WT and mutant p53 proteins. **g** Enrichment profile of de novo binding motifs for WT and mutant p53 proteins are shown. The color represents −log10 (hypergeometric test *p*). The detailed ChIP-Seq analysis results can be found in Supplementary Table 3.

When compared to the WT^OE^ cells with 11,277 peaks, fewer peaks were identified for R273C, Y220C, and Y234C cells (733, 1517, and 2145 peaks, respectively), suggesting substantially reduced DNA binding, whereas R273H showed increased DNA binding capacity (25,309 peaks) (Supplementary Fig. 16a). It was particularly interesting that two mutations at the same DNA-contacting residue, R273C and R273H, resulted in contrasting changes in DNA binding capacity, which may underlie their opposite phenotypic trends. Only 6.7% and 4.7% of peaks for R273C and R273H, respectively, were identified within the promoter regions (defined as −2500 to +100 bps of the transcription start sites) (Supplementary Fig. 16b), markedly less than the fractions observed in Y220C (17.3%), Y234C (21.9%), WT^OE^ (18.3%) as well as the entire human genome (8.7%). These mutations at R273 apparently distinguish the DNA binding capacity from the sequence-specific recognition within promoters, especially R273H, which binds to more sites but not specifically. All four mutant p53 proteins, particularly R273C and Y220C, lost promoter binding to native transcriptional target genes of WT p53 and bound to alternative genes (Fig. 6a and Supplementary Table 3a) with a limited overlap between them (Supplementary Fig. 16c). Pathway enrichment analysis (hypergeometric test) on the promoter-bound genes demonstrated that the target genes of p53 mutants shared only a small fraction of enriched pathways for the WT p53 (Fig. 6b), implying a major alteration in downstream cellular events. Overall, the weakly aggressive mutants, Y234C and R273H, shared significantly more common pathways (Chi-squared test *p* < 1.0e−10) with WT than the aggressive Y220C and R273C mutants. When we examined correlation between the pathway enrichments and invasiveness, a total of 52 pathways showed significant inverse correlation with invasiveness (Pearson’s correlation *p* value < 0.05, Fig. 6c and Supplementary Table 3b). Not surprisingly, given the loss of DNA binding in the more invasive cells, no pathways were more enriched in invasive cells. The identified pathways included the Hippo Pathway, implying transcriptional dysregulation by more invasive p53 mutants. Demonstrating loss of binding to known p53-targeted genes, p53/DNA damage/stress response-related pathways were depleted in more invasive Y22C and R273C cells. Other invasion-associated pathways were related to Wnt signaling, nuclear receptor signaling, and intracellular trafficking. Together, these results indicate that loss of binding to canonical p53 targets by mutant p53 proteins and the concomitant neomorphic gain of DNA binding preference may be contributing mechanisms to heterogeneity in invasiveness induced by different missense mutant p53 proteins.

Consistent with the absence of p53-binding inducers, we observed the canonical p53 binding motif of RRRCWWGYYY^68^ in only 15.5% of all identified peaks even for the WT^OE^ cells. This agrees with a previous study of WT p53 in the absence of any stress stimuli^69^ but was less than 30% for other studies that used p53-inducing conditions^67^. Nonetheless, the p53-binding motif sequence was significantly enriched for the WT p53 (hypergeometric test *p* = 1.0e-9) followed by R273H (hypergeometric test *p* = 1.0e-7), but not for other mutants (Fig. 6d). Similarly, well-known targets of WT p53 such as *CDKN1A* were bound by only p53 WT^OE^ and R273H but not the other p53 mutants (Supplementary Table 3a). When enrichment of all known transcription factor binding motifs were compared, R273H and WT^OE^ cells showed a similar profile, while other mutants had reduced levels of motif enrichment (Fig. 6e, Supplementary Fig. 16e, and Supplementary Table 3c). Notably, the R273C mutant almost lost overall DNA binding capacity (Fig. 6a) but appeared to gain binding to a new motif, such as the CUX1 motif (Fig. 6e). Further, a de novo motif discovery analysis showed that a total of 101 motifs (FDR < 0.05) were identified across all cell lines, and R273C and R273H bound to fewer motifs than WT, Y220C, and Y234C (Fig. 6f and Supplementary Table 3d). When the cell lines were clustered based on the enrichment levels of de novo motifs, R273C showed the most distinct profile from that of WT p53 (Fig. 6f, g). Overall, our ChIP-Seq analysis demonstrated that R273H retained similar DNA binding capability and preference as the WT p53, while the R273C was dissimilar.

### Transcriptional regulatory function of different p53 missense mutants inferred by integrated analysis of RNA-Seq and ChIP-Seq data

**Fig. 7:**
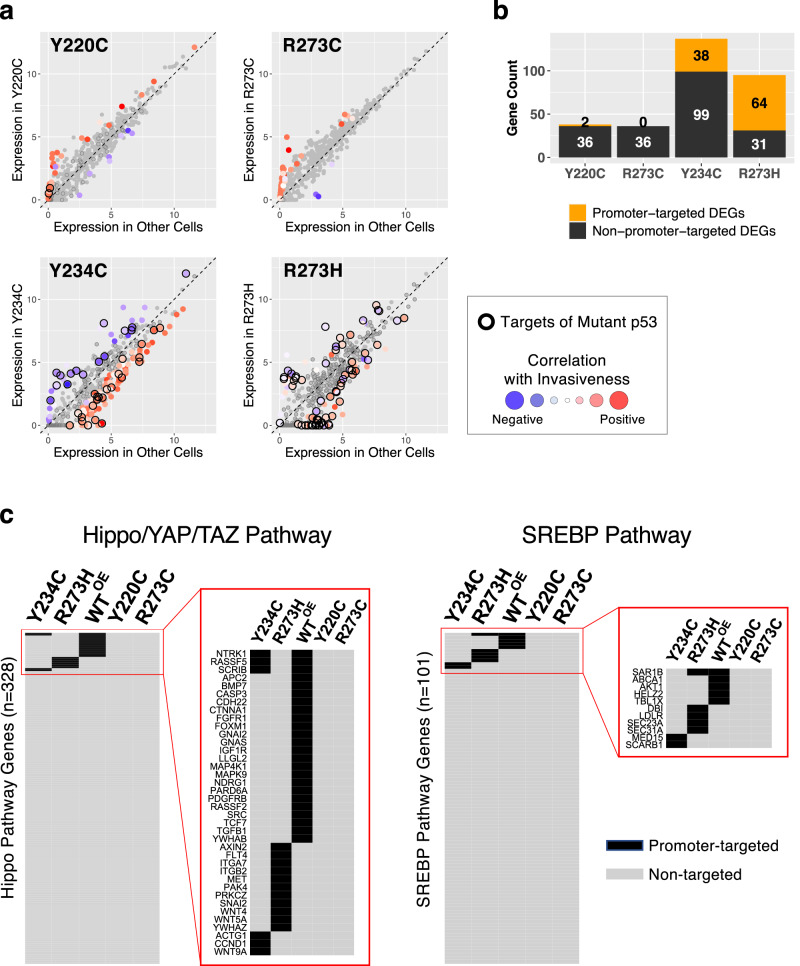
Integrated analysis on RNA-Seq and ChIP-Seq. **a** Gene expression levels, shown as log2(TPM), in each p53 mutant cell line (y axis) were compared to the mean expression levels in the other 9 mutant p53 expressing cell lines (*x* axis), and the differentially expressed genes (DEG, *Z* score-converted *q* < 0.05) were color-coded according to the correlation between expression levels and invasiveness across all cell lines. To examine the impact of p53 binding on the direction and the degree of expressional changes, the promoter-targeted genes identified by ChIP-Seq for each p53 mutant are highlighted with black border. **b** The numbers of promoter-targeted (in orange) and non-targeted (black) DEGs by each p53 mutant are shown. **c** Promoter-targeted genes (peak detection *q* < 0.05, in black) by WT and mutant p53 proteins in the Hippo/YAP/TAZ and the SREBP pathways are displayed as heat maps.

To estimate the contribution of p53 mutant protein to altered gene expression, we integrated the RNA-Seq and ChIP-Seq data and quantified the fraction of the differentially expressed genes (DEGs) that were promoter-bound by the p53 mutant protein (Fig. 7a). As p53 functions as both a direct transcriptional activator and an indirect transcriptional repressor^70^, the direction of differential expression was also examined. The mutants R273H and Y234C induced both positive and negative gene expression changes, of which a large fraction (64/95 and 38/137 genes, respectively) corresponded to direct promoter-binding targets of the mutant p53 forms (indicated by a black ring). In contrast, the invasive p53 mutants, R273C and Y220C, were biased towards upregulated genes, and very few of the mutant DEGs were promoter binding targets, indicating an indirect mode of transcriptional regulation (Fig. 7b).

As the Hippo/YAP/TAZ and the SREBP pathways were identified as the top invasion-associated pathways, we examined whether the genes in these pathways were directly targeted by mutant p53 proteins. Interestingly, all four p53 mutants lost promoter binding to all or most genes in the pathways that were normally bound by WT p53 such as *PDGFRB* and *AKT1*, but less invasive mutants Y234C and R273H gained binding to a few genes that were not bound by WT p53 (Fig. 7c), implying pathway dysregulation and consequent phenotypic alteration.

Examination of individual target genes of mutant p53 proteins provided insights on the potential regulatory action of each mutant. For example, *TFAP2A* was a binding target of the least invasive Y234C mutant and displayed a twofold increase in mRNA expression in Y234C cells, in agreement with a report that overexpression of this gene associates with less cell migration and invasion^71^. *CASZ1* gene promoter was bound only by R273H, and the expression was specifically abolished in R273H cells. *CASZ1* encodes a zinc finger transcription factor and a tumor suppressor^72,73^, and the somatic mutations in breast cancer were associated with poor survival (Supplementary Fig. 17).

In summary, our cancer phenotype characterization and integrated analysis at the molecular level demonstrate heterogeneous cellular phenotypes induced by different p53 missense mutant proteins that can be attributed in part to the mutant-specific changes in downstream target gene expression via both direct and indirect regulatory mechanisms by altered DNA binding capability and preference.

## Discussion

**Fig. 8:**
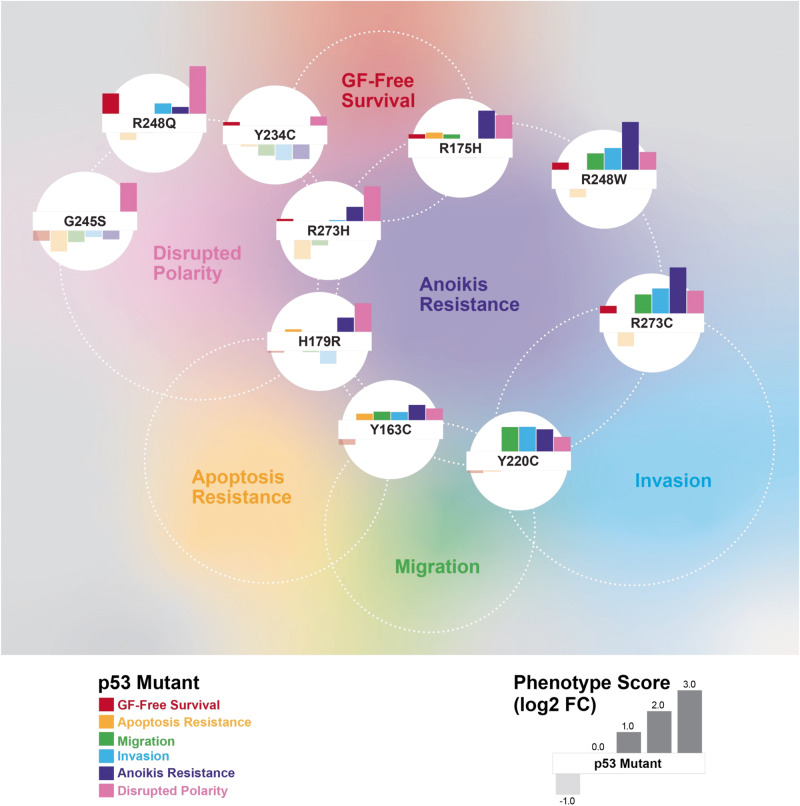
Overview of heterogeneous functional impact of different p53 missense mutations on cancer hallmark phenotypes. The normalized phenotypic scores (as log2 fold changes over controls WT^OE^ cells as shown in the legend) for each mutant p53 protein-expressing cells are shown as bar plots. The cell lines were positioned by their relative phenotypic profiles calculated by PCA, while the positions of phenotype names are approximately centered among the cells with more aggressive behavior.

More than 100 different missense mutations occurring in *TP53* may drive the heterogenous behavior of TNBC tumors. To explore these in a consistent cellular background, we generated stable MCF10A cell lines expressing the ten most common missense mutant p53 proteins in breast cancer. Based on the observed levels of mutant proteins (Supplementary Fig. 1b), the p53 tetramers in the cell lines predominantly function as mutant p53. The quantified phenotype scores for six cancer-like cellular behaviors for each cell line (compared to control WT^OE^ cells) were compared to their gene expression profiles and promoter binding by the mutant proteins and inferred pathway-level mechanisms underlying the heterogeneity. By not stimulating or provoking the cell lines, except doxorubicin treatment to measure apoptotic resistance, our assay results likely captured the functional impact of the founding driver mutations in *TP53* close to physiological conditions at the early stage of breast cancer development in vivo. The mutant p53-expressing cell panel showed a range of phenotypic differences via gain-of-function, distinct from the loss-of-function phenotypes of WT^KD^ cells, and the direction and the extent of the changes by each mutant was highly variable (Figs. 3a and 8). This reflects that the mutations affect different cellular functions of p53 to varying degree. It should be noted that the summarized or averaged phenotypic aggressiveness (Fig. 3a) was primarily to describe the relative differences in phenotypes within the tested cell lines in the context of phenotypic heterogeneity. The absolute levels of aggressiveness would otherwise require a large comparative study with cancer cells with a broad range of well-defined aggressiveness for each phenotype. Notably, no obvious trend distinguishing the conventional DNA contact and the structural mutants was observed throughout the study.

Compared to breast cancer cell lines with multiple oncogenic mutations that have been used to study cellular impacts of *TP53* mutations, the isogenic MCF10A cell line model used in this study is highly informative in measuring the p53 missense mutation-specific changes without confounding interference from other genetic and epigenetic alternations, although we fully acknowledge that single cell line models have clear limitations in understanding the full spectrum of functional impacts of *TP53* mutations in the context of highly heterogenous TNBC. Importantly, the heterogeneous phenotypic changes induced by the ten different p53 mutants in this study correlated well with overall survival of the basal-like breast cancer patients (Fig. 3b, c, and Supplementary Fig. 10), which supports the clinical relevance of our MCF10A model system for studying cellular and molecular mechanisms of the phenotypic heterogeneity. The results also suggest that the type of *TP53* missense mutations acquired at the very early stage of cancer development may predispose breast tumors to more aggressive clinical phenotypes. In agreement, the invasive R273C and Y220C mutants showed comparable levels of invasiveness to that of metastatic MDA-MD-231 cells (Supplementary Fig. 6) with a *TP53* mutation (R280K) and other oncogenic mutations in *BRAF*, *KRAS*, and *TERT* genes. However, when we xenografted the aggressive R273C, R248W, and Y220C cells and the less aggressive Y234C cells (*n* = 2 each) in the NOD/SCID mice, no detectable tumor was observed up to 10 months post injection, whereas the control MDA-MB-231 cells formed tumors that metastasized to the lung and the liver within 2 months (*unpublished results*), agreeing that *TP53* mutation alone is not sufficient to enable tumor formation in vivo^74,75^. Gaining a partial set of cancer hallmark phenotypes in vitro from *TP53* missense mutation is not sufficient for tumor formation in vivo, which requires additional mutations for aggressive phenotypes. We are currently performing genome-wide screening to find such additional mutations that can drive TNBC development in collaboration with specific missense *TP53* mutations,

In our study, the p53 mutants R248W, R273C, Y220C, and R248Q showed more aggressive phenotypes across all cancer hallmarks (Fig. 3a). R248Q and R248W mutants were resistant to doxorubicin-induced apoptosis, and, in locally advanced breast cancer patients receiving doxorubicin monotherapy, point mutations at the R248 residue were implicated in primary resistance to doxorubicin and early relapse in patients^76^. Li-Fraumeni Syndrome (LFS) patients with germline mutations at position R248 showed faster tumor onset and worse survival than those at G245^77^. The expression of the R248W mutant in p53-null H1299 lung carcinoma cells stabilized Slug, a driver of EMT, to promote cell invasion^78^, a behavior also observed in our study. In line with our results (Fig. 2, Supplementary Figs. 8 and 9), earlier work showed that knock-down of endogenous WT p53 in MCF10A cells led to formation of mammary acini with partially cleared lumen and altered staining of β-catenin and laminin V, whereas ectopic expression of R248W led to disrupted acini with filled lumen and increased expression of mesenchymal markers^49^. Further, in agreement with our survival analysis results (Fig. 3b and Supplementary Fig. 10), in a report on 1794 women with primary breast cancer^79^, the R248W mutation (*n* = 8) was associated with the poorest prognosis compared to any other missense mutations, and missense mutations at R248 residue were also associated significantly with shorter survival than the other *TP53* hotspot mutations^80^. In a recent in-vivo study^81^, mice with the somatic mutation *Trp53*^R245W/+^ (R248W in human) produced the most aggressive and metastatic breast tumors with 46% metastasizing early to the lung and the liver, whereas R172H (R175H in human)-mutated tumors were less aggressive, and only a small fraction disseminated to lungs.

The phenotypic effects of R273H, G245S, and Y234C mutants were either similar to or weaker than those of WT^KD^ (Fig. 3a). G245S was the least aggressive of all mutants, where the only notable cancer-like behavior was its formation of dense and depolarized mammospheres, consistent with previous observations^49^. In contrast to R273C, R273H cells were apoptosis-sensitive and less migratory and invasive, which agrees with a report that the mutant R273H cooperated with p21 to promote Slug protein degradation decreasing cell invasion^82^. The R273H cells were resistant to anoikis, like the breast cancer cell line MDA-MB-468 harboring R273H mutation that resisted anoikis^83^. Breast cancer patients with G245S mutation had better survival^79^ than the patients with R248W mutation. Moreover, in LFS patients the G245 mutation associated with significantly delayed tumor onset^80^. Despite exhibiting a reduced DNA binding activity than WT p53, the G245S mutant still binds to the *Gadd45* promoter^84^. The G245S mutation was shown to destabilize p53 protein only by <2 kcal/mol relative to WT p53, compared to >3 kcal/mol by R175H mutation^85^. In agreement, our molecular dynamics simulations showed similar DNA binding dynamics for mutant G245S (G242S in mouse) and the WT p53 protein, wherein the number of protein residues in contact with DNA was unchanged between WT and G245S (Supplementary Fig. 18a, b). Both mutations G245S and R273H introduce only localized changes in the protein conformation and preserve the tertiary folding found in WT p53^86^, providing explanation for their limited impact on cellular phenotypes. The rest of the p53 mutants Y163C, R175H, and H179R showed moderately aggressive phenotypes comparable to that of WT^KD^. Though the R175H protein was most abundantly expressed (Supplementary Fig. 1b), the elevated mutant protein levels didn’t lead to more aggressive phenotypes.

Integration of phenotype assays with the RNA-Seq and ChIP-Seq results provided multi-level molecular profiles for inferring functional impacts of different *TP53* missense mutations. Taking the advantage of our quantitative multi-dimensional phenotypic and transcriptomics data, we developed a comprehensive pathway analysis pipeline utilizing three complementary computational methods to interrogate linear relationships between the pathway-wise gene expression matrix and individual phenotype vectors and collectively identify the Hippo pathway as the top term associated with aggressive phenotypes including GF-free survival, resistance to apoptosis, cell invasion, and disrupted mammosphere polarity (Fig. 4). A large fraction of genes in the pathway and the downstream signature genes of YAP/TAZ activation were elevated in more invasive p53 mutant cell lines (Fig. 5c and Supplementary Fig. 13), in agreement with its roles in cell proliferation, stemness, EMT, and drug resistance in breast cancer^57–59,87^. Mutant p53 proteins and YAP/TAZ promote tumorigenesis^61,88,89^ via direct interaction^90,91^ or mediated by LATS2/MDM2^61^. The SREBP/mevalonate pathway, another top pathway associated with migration/invasion (Fig. 4), is also functionally linked to the Hippo/YAP/TAZ pathway and *TP53* mutations. Mutant p53 proteins (e.g., K280R) are known to function as coactivators of SREBP-mediated transcription to turn on the mevalonate pathway and the Rho GTPases, the upstream activators of oncogenic YAP/TAZ functions^60^. WT p53 blocks maturation of SREBP2, thus, repressing the mevalonate pathway^92^, but mutant p53 upregulates the mevalonate pathway by interacting with mature SREBP proteins^93^. Validating the in-silico pathway analysis results, promoter binding to the Hippo and SREBP pathway genes were significantly impaired in more invasive cells (Figs. 6c, 7c), and a significant correlation was observed between the invasiveness and the activity of TEAD, the downstream transcriptional effector protein family of the Hippo pathway (Fig. 5d). Further, the elevated Hippo pathway signatures were strongly associated, even stronger than the p53 pathway, with the aggressive basal-like breast cancer subtype in both cell lines and tumors (Supplementary Fig. 14) and with poor survival among the patients with basal-like tumors (Supplementary Fig. 15b), indicating that the Hippo/YAP/TAZ signature may be a strong indicator or predictor of clinical phenotypic aggressiveness in early-stage tumors and a potential therapeutic target for early intervention.

Although YAP1 and TAZ proteins are considered functionally redundant, there are reports of contextual dominance of one or the other in certain settings. In our model system, only TAZ was regulated concordantly with invasiveness through elevated mRNA levels in more invasive cell lines (Fig. 5c and Supplementary Fig. 13). In agreement, TAZ protein but not YAP1 was more abundantly expressed in highly invasive breast cancer cell lines^62^, and only TAZ mRNA was significantly elevated in TNBC tumors over the ER-positive tumors^63^. Directly relevant to our study, TAZ but not YAP1 was elevated in Ras-transformed MCF10A cells that formed high-grade tumors in mice^57^, and overexpression of TAZ in MCF10A cells increased cell invasion^62^. Further, hyperactivation of TAZ but not YAP1 was shown to play a pivotal role in onset of basal-like breast cancer in mouse model, and concomitant p53 knock-out accelerated the process^94^. These collectively suggest that different missense mutant p53 proteins lead to various levels of invasiveness via differential regulation of the Hippo/TAZ axis.

Our data showed that changes in a single amino acid resulted in remarkable changes in the phenotype as well as the molecular properties. For example, two different mutations at DNA-contacting R273 residue displayed a striking difference in phenotypic outcomes (Fig. 3a) and DNA binding (Fig. 6). The less aggressive R273H mutant retained p53 motif-specific as well as overall DNA binding capacity, while more aggressive R273C showed near complete loss of DNA binding, potentially due to the replacement of Arg with the smaller Cys. In agreement, R270H mutation in mice (R273H in human) displayed weaker dominant negative effects than other p53 mutants^43^. In MD simulation, both R273C and R273H mutants showed fewer protein-DNA contacts and weaker DNA interactions compared to WT p53 (Supplementary Fig. 18c). Though R273H showed some electrostatic interaction with DNA that weakens over time, no such interaction was seen for R273C. The number of R273H-DNA contacts reduced slowly from 24 and stabilized to 6 over 100 ns during the simulation. This reduction in contact was much more drastic for R273C, reducing to two contacts almost instantly, and most of the simulated models did not show any DNA contact of this residue. Analysis of intermolecular interaction energies revealed that R273H-DNA contact was mediated by electrostatic interactions of the positively charged histidine with the nitrogen and oxygen atoms of thymine 18 (Supplementary Fig. 18c). These electrostatic interactions were eliminated in the R273C mutant, which resulted in significantly lower interactions with the DNA. Of note, the native arginine, with a higher pKa than histidine, formed stronger contacts with the DNA. In addition, R273H had a subtle effect on the thermodynamic stability and a half-life like that of WT p53, whereas R175H caused strong structural perturbation destabilizing the core domain^95^.

Though DNA contact mutations can alter crucial DNA contact points without introducing large perturbations in overall structure and DNA-binding surface, replacement with a large hydrophobic side chain could prevent sequence-specific DNA binding. For example, R248W with a hydrophobic tryptophan affected the cancer phenotypes more severely than R248Q, with a polar and positively charged glutamine. Surprisingly, mutant R248W showed increased protein–DNA contact compared to R248Q through pi-stacking interactions as deduced from MD simulation data (Supplementary Fig. 18d). Though both mutants R248W and R248Q showed fewer protein-DNA contacts than WT p53, the protein–DNA interaction changes are not as contrasting as R273H and R273C mutants. At the G245 residue, substitution of glycine with serine (G245S) does not severely impair the tertiary structure of p53, and the L3 loop is conserved (Supplementary Fig. 18a), which may explain why mutant G245S produced the least aggressive phenotypes. In contrast, introduction of the bulky histidine at R175 caused structural distortions and disrupted the zinc-binding pocket of p53^96^, which might lead to moderately aggressive phenotypes as in our study. Substitution of hydrophobic tyrosine with small cysteine residue (Y163C, Y220C, and Y234C) created highly heterogeneous phenotypes. The Y220C mutant protein was reported to contain a large surface crevice and perturb the packing of the β-sandwich^96^, but our MD simulation data did not show a significant change in protein-DNA interactions. This is because the residue is located far (~30 Å) from the protein-DNA surface, and the simulation times needed to observe allosteric effects over such long distances are prohibitively high. Overall, we observed a good correlation between the deviation in structure and the function (phenotypes) of missense p53 mutant proteins.

## Methods

### Production of MCF10A cell lines expressing p53 mutant proteins

The MCF10A cells (ATCC, CRL-10317) expressing WT and missense mutant p53 proteins were made using the Gateway plasmids generated by plasmid repository DNASU (dnasu.org). The *TP53* gene inserts with missense mutations were transferred into pLX304 (for WT p53, Addgene, # 25890) or pLenti4/V5-DEST vectors (for missense mutants, ThermoFisher Scientific), and stable cell lines were prepared through lentiviral transduction. DNASU clone IDs for the plasmids are WT-p53 (HsCD00435064), Y163C (HsCD00966033), R175H (HsCD00966046), H179R (HsCD00966059), Y220C (HsCD00966058), Y234C (HsCD00966070), G245S (HsCD00966022), R248Q (HsCD00966071), R248W (HsCD00966065), R273C (HsCD00966038), and R273H (HsCD00966072).

### Cell culture

MCF10A cells (female mammary epithelial cells), including WT and mutant 53-expressing cells, were cultured at 37 °C in a 5% CO_2_ humidified incubator. DMEM-F12 media (ThermoFisher #11320-082) was supplemented with 5% Horse Serum (ThermoFisher #16050-122), hEGF (10 ng/ml, ThermoFisher #PHG0311), hydrocortisone (0.5 µg/ml, Sigma #H0888), cholera toxin (100 ng/ml, Sigma #C8052), and Insulin (10 µg/ml, Sigma #I9278). Cells were routinely passaged with trypsin (0.25% in HBSS with 0.2 g/ml EDTA, GE Healthcare #SH30042.01) at 80–90% confluence. MCF10A cells were obtained from ATCC (CRL-10317, RRID:CVCL_0598), and cell authentication has not been performed. The cells were checked for mycoplasma infection at regular intervals. The experiments were performed using cells with limited passage numbers and fresh cell aliquots were used for replicate experiments.

### Western blot

Cells were grown to 80% confluency in six-well plates (Greiner #5665-7160) and lysed using RIPA lysis buffer containing 50 mM Tris, 150 mM NaCl, 1% IGEPAL CA-630 (Sigma #I9996), 0.1% sodium azide (Sigma #S8032), cOmplete mini protease inhibitor (Sigma #04693124001) or Halt protease and phosphatase inhibitor cocktail (Thermo Scientific #78442), 200 µM sodium fluoride (Sigma #S6776) and 200 µM of sodium orthovanadate (Sigma #450243). Cells were scraped, incubated on ice for 10 min and centrifuged at 7400 g. Lysates were quantified using the Pierce BCA kit (#23225). Samples were run on 4–20% TGX gels from BioRad (#567-1093) and blotted onto 0.45 µm PVDF membrane (GE Healthcare #10600023) using the BioRad semi-dry transfer system. Primary antibody for p53 protein from Sigma (#P6874) diluted to 1:200 and secondary HRP-linked anti-mouse antibody (Cell Signaling Technologies #7076) diluted to 1:3000 was used to visualize p53 bands. The p53 mutant proteins were seen using the V5 tag Ab (CST, Cat no 13202S). All blot images in each figure were from the same experiment and processed in parallel.

### Cell viability assay

Cells were parallelly plated in clear CellBIND 96-well plates (Corning) for pictures and opaque white plates for chemiluminescent analysis (Perkin-Elmer) and allowed to grow for 24 h in normal growth media before treatment. The following day, media was replaced with the restricted media with or without growth factors, serum and EGF. Human EGF (10 ng/ml, ThermoFisher) and horse serum (5% v/v, ThermoFisher) were excluded individually or together from normal growth media. Cells were grown for 72 h, and cell viability analyzed by Cell Titer-Glo (Promega) on the Envision plate reader (Perkin-Elmer). Results were calculated with respect to untreated cells, then log2 transformed.

### Apoptosis assay

Cells (4000 cells/well) were plated into both 96-well CellBIND clear plates (Corning) for observation and Perkin-Elmer white opaque plates. The following day, cells were treated with 0.3, 0.7, 1.0, or 3.0 µM Doxorubicin (Calbiochem) using the Biomek NX liquid handler (Beckman Coulter) and incubated for 24 h. For evaluating apoptosis induction, cells were treated with Caspase-Glo 3/7 (Promega) per supplier protocol and read in the Envision plate reader (Perkin-Elmer). Results were reported as the area-between dose-response curves of log_2_ fold-change relative to the MCF10A WT^OE^ cells as shown in Supplementary Fig. 3.

### Cell migration and invasion assay

200 µl serum free media was added to the inserts, incubated at 37 °C for at least 1 h to warm the Matrigel coat. 2.5 × 10^5^ cells (in 200 μl) in serum free media were seeded in the upper well of the Matrigel coated (Corning) or non-coated (Corning) cell culture insert for invasion and migration assays, respectively. The media with serum was placed in the lower well. The plates were incubated for 22 h. The migratory or invasive cells were first fixed with 4% paraformaldehyde for 2 min at RT, permeabilized by adding 500 μl of 100% cold methanol (2 min at RT) per well and stained with 0.5% crystal violet. A media-moistened cotton swab was used to remove any cells that did not migrate or invade through the membrane. Cells were imaged under a microscope at 4× and 10× magnification and counted by *CellProfiler*^97^.

### Immunofluorescence

This protocol was used for staining cells in a 96-well (VWR #82050-748) format and a shaker was used during antibody incubations to obtain uniform staining. The cells were washed twice with 1x PBS (100 µl) and fixed with 75 µl of 4% paraformaldehyde (VWR #AA43368-9M) per well (15 min at RT). Cells were washed with PBS and 75 µl of Blocking Buffer (5% Goat Serum Life Technologies #PCN5000 with 0.32% Triton X-100 Sigma #T8787 in 1× PBS) added per well (1 h at RT). After PBS wash, 50 μl/well of primary antibody (at recommended dilutions in Ab protocol) in the Antibody Buffer (1% BSA and 0.3% Triton X-100 in 1x PBS) was added and incubated at RT, shaking gently in an orbital shaker for 1–2 h for same day processing or overnight at 4 °C while shaking. Cells were washed with PBS and 50 μl/well of secondary antibody in Antibody Buffer (at recommended dilutions in Ab protocol) was added with incubation at RT, shaking gently in an orbital shaker for 1–2 h. After final wash in 1x PBS, plates were wrapped in aluminum foil and stored at 4 °C with 100 µl 0.05% Sodium Azide (NaN_3_, Sigma #S8032) in PBS.

### 3D cell invasion assay

Matrigel (BD Matrigel 354230, stored at −20 °C) was thawed while keeping in ice in a 4 °C refrigerator overnight and diluted to 3 mg/ml in ice-cold serum free media. The collagen coated 96-well plate (Platypus tech Cat no. CMACC5.101) was removed from 4 °C and allowed to equilibrate to RT. The underside of the plate was checked to ensure that the seeding stoppers were firmly sealed against the bottom of the plate. 100 µl of suspended cells (80,000 cells per well) were added into each well (control & test wells) without disturbing the seeding stopper, lightly tapping the plate to evenly distribute the cells in each well. The seeded plate with stoppers was incubated at 37 °C and 5% CO_2_ for 6–8 h to permit cell attachment. Using the stopper tool seeding stoppers were removed. Reference or the control wells remained with the stoppers till the end of the assay. Media was removed with a pipette and the well gently washed with 100 µL of serum-free media to remove any unattached cells. 50 µl of the Matrigel overlay was added to each well. The plate was incubated at 37 °C and 5% CO_2_ for 1 h to permit the Matrigel polymerization, and 100 µl of serum-containing media was put on top of the 3D matrix. The plate was incubated at 37 °C for 16 h to permit cell invasion. For end-point analysis cell seeding stoppers were removed from the reference/control wells, Matrigel overlay was added and allowed to form the gel. The cells were stained with 5 µM cell tracker green (100 µl/well, 45 min, 37 °C) and 0.5 uM Vybrant Violet dye (100 µl/ well, 30 min, 37 °C). Wells were washed with 1x PBS and fixed using 3.7% paraformaldehyde in 1x PBS for 15 min at RT. Images were taken with *ImageXpress* at different planes on the *Z* axis.

### Anoikis assay

Cells were plated in low-binding 96-well plates at 150 cells/well and maintained in regular growth media for 7 days. Vybrant Violet Dye (0.5 µM, ThermoFisher Scientific) was added to the cell and incubated at 37 °C for a minimum of 30 min, and Ethidium Homodimer (0.3 µM, Biotium) was then added before analysis. The MetaXpress High Content Image Acquisition platform (Molecular Devices) was used to capture images of a total of eight wells for each cell line, and only the images of the entire cell mass was used for image analysis. ImageXpress image analysis software (Molecular Devices) was used to identify and count the cells stained with either Vybrant Violet (Cy3, blue) or Ethidium Homodimer (Cy5, red). Data was reported as a log2 ratio of live to dead cells (Cy3:Cy5).

### 3D mammosphere culture and analysis

96-well black µClear plates were pre-coated with 10 µl of Matrigel (Corning). Cells were added on top of the Matrigel, cultured for 9 days, and then fixed with 4% paraformaldehyde (Alfa-Aeser) for 15 min and washed with 100 mM Glycine-PBS solution to neutralize the paraformaldehyde, followed by permeabilization with 0.5% Triton X-100 (Sigma-Aldrich) for 15 min at 37 °C. Permeabilized cells were blocked with 10% goat serum (ThermoFisher Scientific) and 20 μg/ml goat α-mouse IgG (Jackson ImmunoResearch) for at least 1 h. The spheroids were washed with PBS, and cell staining was done as previously described^41^. The equatorial cross-section of a minimum of 50 mammospheres per cell line was captured for the quantitative analysis. Analysis was performed by employing custom Cell Profiler pipelines. For identification of spheroids, the β-catenin staining was used as a mask. The area of spheroids was determined by measuring the diameter of cross-sections at the equatorial plane. Intensity measurements were based on the average pixel intensity over the object mask for each target protein. To analyze distribution of laminin (cell polarity) and the nucleus (clearing of lumen), concentric rings/bins were created from the center of each object, and the average pixel intensity per ring was measured.

### Survival analysis

Two clinical breast cancer datasets, TCGA^51^ and METABRIC^52^, were obtained from cBioPortal^98^, and the subset of patients labeled as “*basal-like*” were selected. Samples with the 10 *TP53* missense mutations used in this study were grouped by the mutation type, whereas the samples without any *TP53* mutations are considered as the wild-type (WT) group. The 5-year overall survival rates for the sample groups were analyzed by the *Survminer* (v0.4.9) *R* package. Mutation groups with fewer than three samples were excluded.

### RNA-Seq

Total RNA extracted from the cells (Qiagen cat no. 74134) were used to prepare cDNA using Nugen’s Ovation RNA-Seq System via single primer isothermal amplification (Catalogue # 7102-A01) automated on the Apollo 324 liquid handler from Wafergen. cDNA was sheared to ~300 bp fragments using the Covaris M220 ultrasonicator. Libraries were generated using Kapa Biosystem’s library preparation kit (KK8201). Fragments were end-repaired and A-tailed, individual indexes and adapters (Bioo Scientific, catalogue #520999) were ligated on each separate sample. The adapter ligated molecules were cleaned using AMPure beads (Agencourt Bioscience/Beckman Coulter, A63883), and amplified with Kapa’s HIFI enzyme. The library was then analyzed on an Agilent Bioanalyzer, and quantified by qPCR (KAPA Library Quantification Kit, KK4835) before multiplex pooling and sequencing (2 × 75 bps, paired end) on the NextSeq500 platform (Illumina) at the ASU’s Genomics Core facility.

### RNA-Seq data processing

Raw sequencing read data quality were analyzed using *FastQC* (v0.10.1). STAR (v020201)^99^ was used to align reads to the Ensembl human genome (GRCh38.p14/hg38, release 92) to counts, as shown below.

*STAR --genomeDir genome/ --quantMode GeneCounts --readFilesIn cell_1_R1.fastq cell_1_R2.fastq –outFileNamePrefix cell_1 --outFilterType BySJout --outFilterMultimapNmax 20 --alignSJoverhangMin 8 --alignSJDBoverhangMin 1 --outFilterMismatchNmax 999 --outFilterMismatchNoverReadLmax 0.04 --alignIntronMin 20 --alignIntronMax 1000000 --alignMatesGapMax 1000000*

Duplication rate was obtained, and duplicated reads were marked by *PICARD* (v2.18.3), as shown below.

*java -jar picard.jar MarkDuplicates --INPUT=cell_1.bam --OUTPUT=marked_duplicates_cell_1.bam --METRICS_FILE=marked_dup_metrics_cell_1.txt --REMOVE_DUPLICATES=false*

Duplication plots were generated using R package *dupRadar* (v1.20.0)^100^. As high duplication rates were observed (85.81–97.24%), gene counts after removal of duplicated reads were used as a reference to check whether the expression levels were overestimated due to PCR duplication. Next, because mitochondrial RNA occupied a large but variable portion of RNA pool in every sample (40–80% of all sequence reads), to avoid the bias in calculating the total read-normalized transcript abundance, mitochondrial RNA data were dropped. Finally, sequencing counts were normalized to TPM (transcripts per million reads).

### Enrichment and model-based pathway analysis

For quantitative pathway analysis, KEGG^101^, Reactome^102^, WikiPathways^103^, and the “*Oncogenic Signature*” gene sets (dysregulated genes upon experimental perturbation of cancer-related genes) from the MSigDB^104^ were utilized. GSEA and ssGSEA were performed with the *gseapy* software (v0.10.4)^105^. PLSR analysis was done using *scikit-learn* (v0.23.2) package with fivefold cross-validation (CV), and gene expression values or ssGSEA scores of the pathway gene sets across 13 cell lines were used for model training with the phenotype scores of the cell lines for each phenotype as targeting vectors. Pearson’s correlation coefficients between the model-predicted and the observed phenotypes were calculated to evaluate the performance of the models for each pathway. Forward feature selection was performed with the *SequentialFeatureSelector* function in *scikit-learn* package with fivefold CV. All plots were drawn by the *matplotlib* library (v3.2.1). While GSEA and ssGSEA utilize global rank-based statistics on the entire genes to estimate the enrichment of a pathway gene set, PLSR performs dimensional reduction and regression on both gene expression and phenotypic spaces to find latent variables (i.e., linear combinations of genes) to maximize the covariance. The PLS1 method was used in this study for modeling each phenotypic vector. Each gene is weighted differently in PLSR models, thus capable of detecting the pathway-to-phenotype association when only a subset of genes is expressed correlatively with phenotypes.

GSEA analysis on phenotype-correlated genes for each phenotype and pathway gene set was performed as below by using a data frame with expression-to-phenotype correlation values for 13 cell lines.

*data* = *gseapy.prerank(rnk=correlation_data_frame, gene_sets* = *”pathway.gmt”*, permutation_num = 10000, min_size = 10, seed = 1)

ssGSEA analysis on RNA-Seq gene expression data for each phenotype and pathway gene set was performed as below by using a data frame with gene expression values of 13 cell lines as well as cells/samples in CCLE^64^, TCGA^51^, and METABRIC^52^ datasets.

*data* = *gseapy.ssgsea(data=gene_expression_data_frame*, gene_sets = ”pathway.gmt”, min_size = 10)

PLSR regression on RNA-Seq gene expression data for each phenotype and pathway gene set was performed as shown below as pseudocode by using a matrix with gene expression values of 13 cell lines and an array with corresponding phenotypic values. Model training and cross-validation was performed with a range of 2–5 components, and the results for the number of components with the best performance (i.e., with the largest correlation values between predicted and observed phenotype) in fivefold CV.

*cv* *=* *sklearn.model_selection.RepeatedKFold(n_splits* = *5, n_repeats* = *5*, random_state = 1)

*X = gene_expression_matrix*

*y = phenotype_array*

*for train_data, test_data in cv.split(X, y):*

*model = PLSRegression (ncomps* = *n_components)*

*model.fit(train_data, phenotype_array)*

*predicted_phenotype = model.predict(test_data)*

### TEAD reporter assay

On Day 1 of this experiment, 100 μL of cells from the thirteen cell lines was plated into a 96-well white plate in triplicates. On Day 2, cells were transfected with the 8xGTIIC-luciferase plasmid with eight TEAD binding motifs (Addgene)^106^, in parallel with the positive control pLX313-Renila luciferase plasmid (Addgene) in separate wells, by using 10 μL of Lipofectamine™ 3000 (ThermoFisher Scientific). After 4 h, the cells were washed twice with PBS and replaced with 100 μL of complete media to prevent toxicity of the Lipofectamine™ 3000 reagent to the cells. On Day 3, 100 μL of ONE-GLO™ luciferase assay reagent (Promega) was added into the experimental test wells and 100 μL of Renilla-Glo® luciferase was added into the positive control wells, and the luminescence of the samples were measured using a luminometer.

### ChIP-Seq

All the MCF10A cell lines were fixed with 1% formaldehyde in PBS and incubated for 10 min at RT. Formaldehyde was quenched with glycine (125 mM final conc.) for 5 min at RT. Cells were rinsed with ice cold PBS twice and scraped with 2 ml of cold PBS with protease inhibitor (Sigma Aldrich Cat no. 04693124001). Cells were washed two more times with cold PBS with protease inhibitor and pellets from 20 million cells were snap frozen and stored at −80 °C. 1 ml of ChIP lysis buffer (1% SDS,10 mM EDTA, 50 mM Tris-HCl pH8.1) with protease inhibitors was added and the cells were sonicated in the Covaris M220 ultrasonicator for 8 min. The time and setting for sonication would vary with the cell-line and concentration. The lysates were spun at maximum speed at 4 °C for 10 min and the DNA concentration was checked. The supernatant was transferred into fresh tube. 25–50 μl of the lysate was incubated at 65 °C overnight with 0.1 M NaCl and run on a 2% agarose gel to check the sonication efficiency. You should get an average fragmentation of ~500 bps. For IP, 30 μl of beads (Thermo Fisher Scientific, Cat no. 11203D) were first washed with 1 ml of cold BSA (5 mg/ml) in PBS at RT (three times). Then V5 antibody (Cell Signaling Technology, Cat no. 13202 S) was added to the beads in 1 ml of PBS + BSA. The beads with the antibody were incubated for 4 to 6 h at 4 °C. The IP was performed on 50 μg of total DNA and the samples were diluted accordingly in the dilution buffer (1% Triton X-100, 2 mM EDTA, 150 mM NaCl, 20 mM Tris-HCl pH 8.0). The diluted chromatin was precleared with 15 μl of washed beads for 1 h at 4 °C. 5% of this precleared lysate was kept as input control. PBS/BSA was aspirated from the antibody coated beads and precleared lysate was added to incubate overnight at 4 °C on a rotating wheel. Next day beads were collected using the magnetic concentrator and washed six times with ChIP RIPA buffer (50 mM HEPES, 1 mM EDTA, 0.7% Na Deoxycholate, 1% NP-40, 0.5 M LiCl pH 7.6) at RT on a rotating platform for 10 min between every wash. Then washed twice with 1X TE (pH 7.6) at RT. 100 μl of Elution buffer (1% SDS, 0.1 M NaHCO3, 0.1 M NaCl) was added to the beads and input samples (make up volume to 100 μl). The beads were vortexed in this solution every few minutes for 30 min in total at RT and incubated at 65 °C O/N for 12–14 h. Next day 1 μl of proteinase K (10 mg/ml) was added and incubated at 42 °C for 2 h. The IP DNA was purified with QIAquick PCR purification kit.

### ChIP-Seq data processing

The total sequencing read counts varied from 43 to 59 million reads per samples. Quality of the reads were analyzed using *FastQC* (v0.10.1). Paired-ended reads were mapped to the reference human genome (GRCh38.p92/hg38) end to end using *Bowtie2* (v2.1.0)^107^.

*bowtie2-build genome.fa genome_bt2*

*bowtie2 -x genome_bt2/ -1 cell_1_R1.fastq.gz -2 cell_1_R2.fastq.gz --mm -S cell_1.sam*

Sam files are converted to bam files and then sorted by Samtools (v1.7)^108^.

*samtools view -bS cell_1.sam > cell_1.bam*

*samtools sort cell_1.bam -o cell_1_sorted.bam*

Non-primary alignment, unmapped reads were removed by *Samtools* (v1.7). Duplication rates were calculated by *PICARD* (v2.18.3) (duplication rate ranges from 18% to 88%) as described above for RNA-Seq. *MACS2* (v2.1.2.20181017) was used for peak calling using BAMPE mode with the *q*-value cutoff of 0.05^109^ by using the input genomic DNA sequencing data as the background.

*macs2 callpeak -t cell_1_IP_sorted.bam -c cell_1_Input_sorted.bam -B -g hs -s 300 --call-summits -q 0.05 -n cell_1 -f BAMPE --outdir outdir*

Peaks were annotated using *Homer* toolkit (Homer v4.9.1)^110^, which was also been utilized for motif finding. Regions located between -2500 to +100 bps of the nearest transcription start sites (TSS) were defined as the promoter region.

*parseGTF.pl genome_annotation.gtf ann -annTSSstartOffset −2500 -annTTSendOffset 2500 > annotations.txt*

*assignGenomeAnnotation annotations.txt annotations.txt -prioritize genome_annotation_promoter2500.txt > stat.txt*

*assignGenomeAnnotation cell_1_peaks.bed genome_annotation_promoter2500.txt -ann annotated_cell_1_peaks.txt*

*annotatePeaks.pl cell_1_summits.bed genome.fa -gtf genome_annotation.gtf > cell_1_distance.txt*

Known and de novo motifs were called by *Homer* toolkit. De novo motifs comparison was done by *Tomtom* in *MEME Suite* (v5.0.1)^111^.

*findMotifsGenome.pl cell_1_promoterPeaks300bpSummit.bed genome.fa outdir/ -size given -len 20 -bg WT-OE_promoterPeaks300bpSummit.bed -mcheck HOCOMOCOv11_full_HUMAN_mono_meme_format.motifs -mknown HOCOMOCOv11_full_HUMAN_mono_meme_format.motifs*

*findMotifsGenome.pl cell_1_promoterPeaks300bpSummit.bed genome.fa outdir/ -mcheck denovo_all_cellLines.motifs -mknown denovo_all_cellLines.motifs*

### Statistical analysis

For comparison of phenotypes between the control WT^OE^ cell line and each mutant p53-expressing cell lines, the single-sample *t*-tests were performed on log2-transformed fold changes over the WT^OE^ to determine if the deviation from zero was significant (*p* < 0.05). Data normality was tested by the Shapiro-Wilk test (*α* = 0.05) with the *scipy* (v1.4.1) Python package for each phenotype and cell, and the majority (75%) of data sets were normally distributed. The means, standard errors, and the sample size for each assay and cell are listed in Supplementary Table 1. To test enrichment of Hippo/YAP/TAZ-related terms in the top phenotype-associated pathways in Fig. 4, GSEA analysis was performed on the ranked list of pathway terms, and the *p* value < 0.05 was used to determine the statistical significance.

### MD simulation

The simulation system was built using the crystal structure of the DNA-binding motif of mouse p53 (PDB ID: 3EXJ)^112^. The system was parameterized with the CHARMM36 force field^113^. The structure was solvated with 24,923 waters to obtain a simulation box of 122 Å × 88 Å × 81 Å. Ten Na^+^ ions were added to neutralize the system. The system was prepared using Visual Molecular Dynamics (VMD) software tool^114^. Mutations were generated using VMD’s PSFGEN plugin. The system was minimized, heated to 300 K, and equilibrated for 2 ns, followed by production runs of 100 or 200 ns. All simulations were performed using the NAMD simulation software^115^. Protein-DNA interactions were quantified by calculating the number of protein-DNA contacts in each frame of the simulation trajectory, where a contact was defined by the number of DNA atoms within 5 Å from the given residue of the protein. For sites that do not directly contact DNA but allosterically control protein-DNA interactions, the effect of mutation was quantified by the number of protein residues in contact with DNA. Analysis of simulation data was performed using VMD.

### Supplementary information

Supplmentary Figure

Supplmentary Table 1

Supplmentary Table 2

Supplmentary Table 3

nr-reporting-summary

## Data Availability

Raw sequencing data for RNA-Seq and ChIP-Seq experiments are available in the NCBI Gene Expression Omnibus (GEO) repository with an accession number of GSE162341. Phenotype data, processed RNA-Seq/ChIP-Seq data, and pathway analysis results used for figures are provided in Supplementary Tables.
