# Supplementary material for: Multidimensional quantitative phenotypic and molecular analysis reveals neomorphic behaviors of p53 missense mutants
Source: NPJ Breast Cancer. 2023 Sep 29;9:78. doi: 10.1038/s41523-023-00582-7 (PMC10541912; doi:10.1038/s41523-023-00582-7)
Supplement: Supplementary file 1 — Supplmentary Figure [file 41523_2023_582_MOESM1_ESM.pdf]

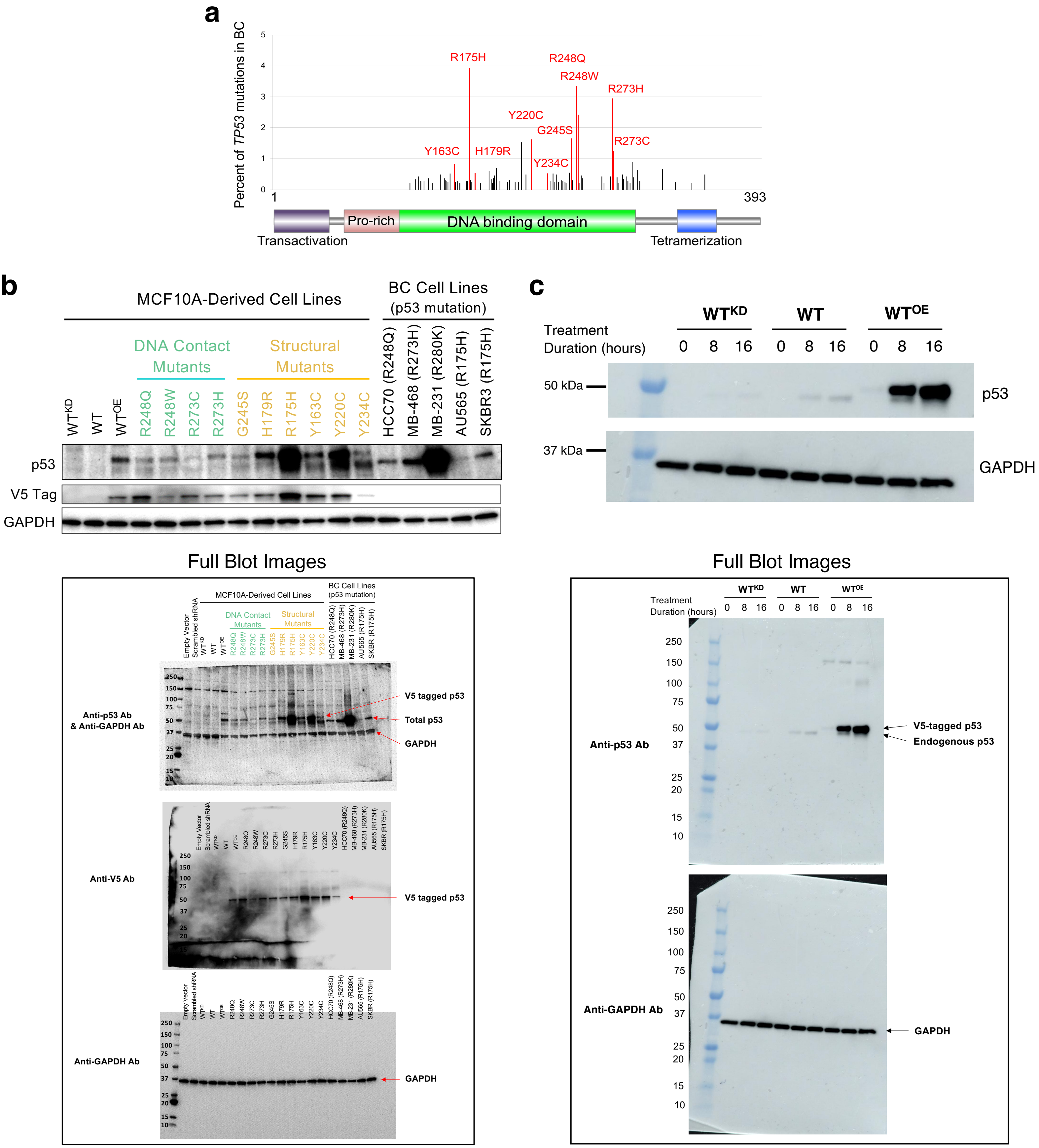

Supplementary Figure 1. Generation of MCF10A-derived cell lines expressing 10 most prevalent p53 missense mutant proteins found in breast cancer. a. Frequent missense mutations (based on the 2012 version of the IARC TP53 Database) are concentrated in the DNA binding domain of p53, which historically have been grouped into DNA contact and structural mutations. Ten mutants utilized in this study are shown in red. **b**. Expression of mutant p53 proteins was confirmed by western blotting. Anti-p53 antibody ("p53") recognizes both exogenous (with V5 tag) and endogenous p53 proteins. As controls, parental MCF10A (WT), p53-knockdown (WT^{KD}), and p53-overexpressing (WT^{OE}) cells were included. The p53 protein levels were also compared with those in other *TP53*-missense mutated breast cancer (BC) cell lines (in green). Original full blot images are shown below. **c**. WT^{KD}, WT (parental MCF10A), and WT^{OE} cells were treated with 1 mM doxorubicin for indicated time, and the p53 protein levels were measured with anti-p53 antibody. Original full blot images are shown below.

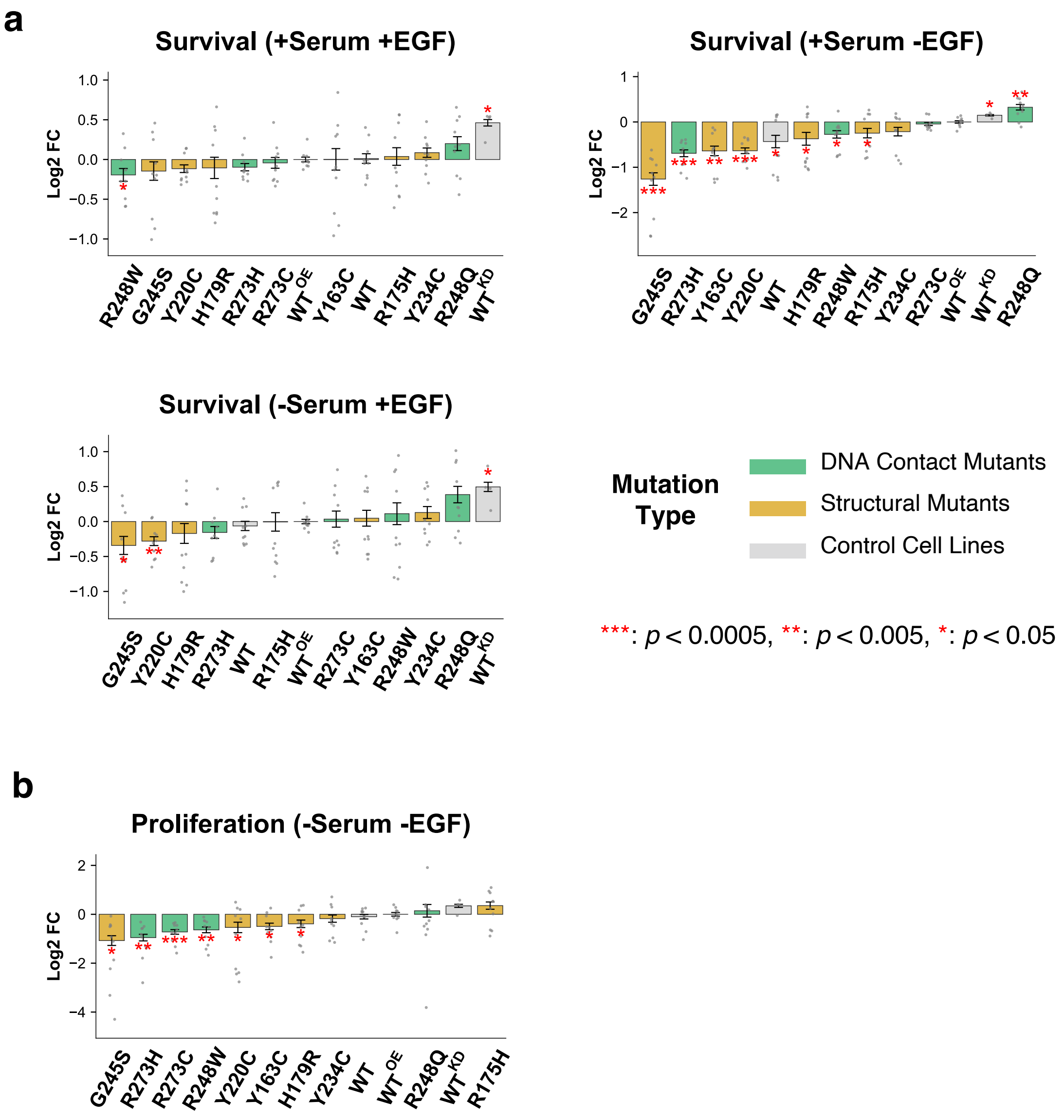

Supplementary Figure 2. Cell viability of MCF10A cell lines expressing different p53 mutants. Survival of cell lines with or without serum and/or EGF were analyzed by three independent experiments, each with triplicates, and the results were normalized to the log2 fold change over the phenotype of p53 WT^{OE} cells. The error bars represent the standard error of mean (SEM) values, and significant differences (two-sided single-sample *t*-test) from the WT^{OE} mean are indicated by red asterisks for indicated *p* values. The p53 mutants are color-coded by mutant type as indicated. In all plots, cell lines were sorted by each phenotype, from less (left) to more (right) aggressive. **a**. Cell viability measured by the CellTiter-glo assay after cells were either grown in the presence of both serum and EGF (+Serum +EGF), in the absence of only EGF (+Serum -EGF), or in the absence of only serum (-Serum +EGF). **b**. Cell counts were measured by DAPI staining (nuclei count) after a 72-hr culture in growth factor-deprived media. Cell lines were sorted by each phenotype, from less (left) to more (right) aggressive.

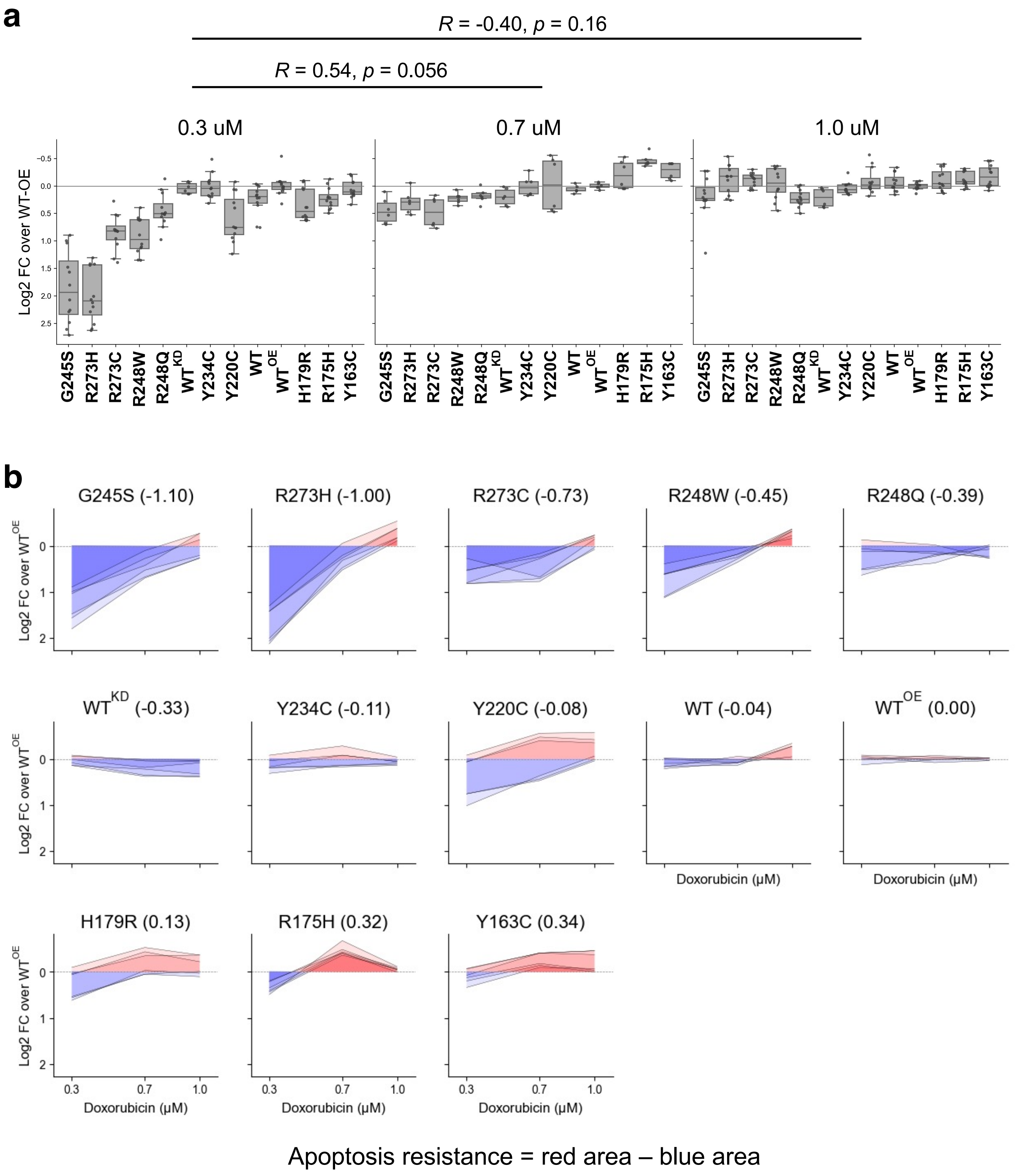

Supplementary Figure 3. Resistance to apoptosis of MCF10A cell lines expressing different p53 mutants. a. The 13-cell line panel was treated with 0.3, 0.7, and 1.0 μM doxorubicin for 24 hours, and the caspase 3/7 activity was measured by the Caspase-Glo 3/7 reagent. The values were then normalized to the log2 fold changes over the WT^{OE} values. To position more resistant values on the top, the Y axis was inverted. Pearson's correlation coefficients and the corresponding *p*-values across the cell lines between 0.3 μM with higher concentrations are shown. Center line: median, box limits: upper and lower quartiles, whiskers: 1.5x interquartile range. **b**. Apoptotic resistance across multiple doses of doxorubicin was summarized by the area between dose-response curves of each cell lines and the WT^{OE} cells. The resistance scores was calculated by subtracting the area of negative values (red shade) by the area of positive values (blue shade).

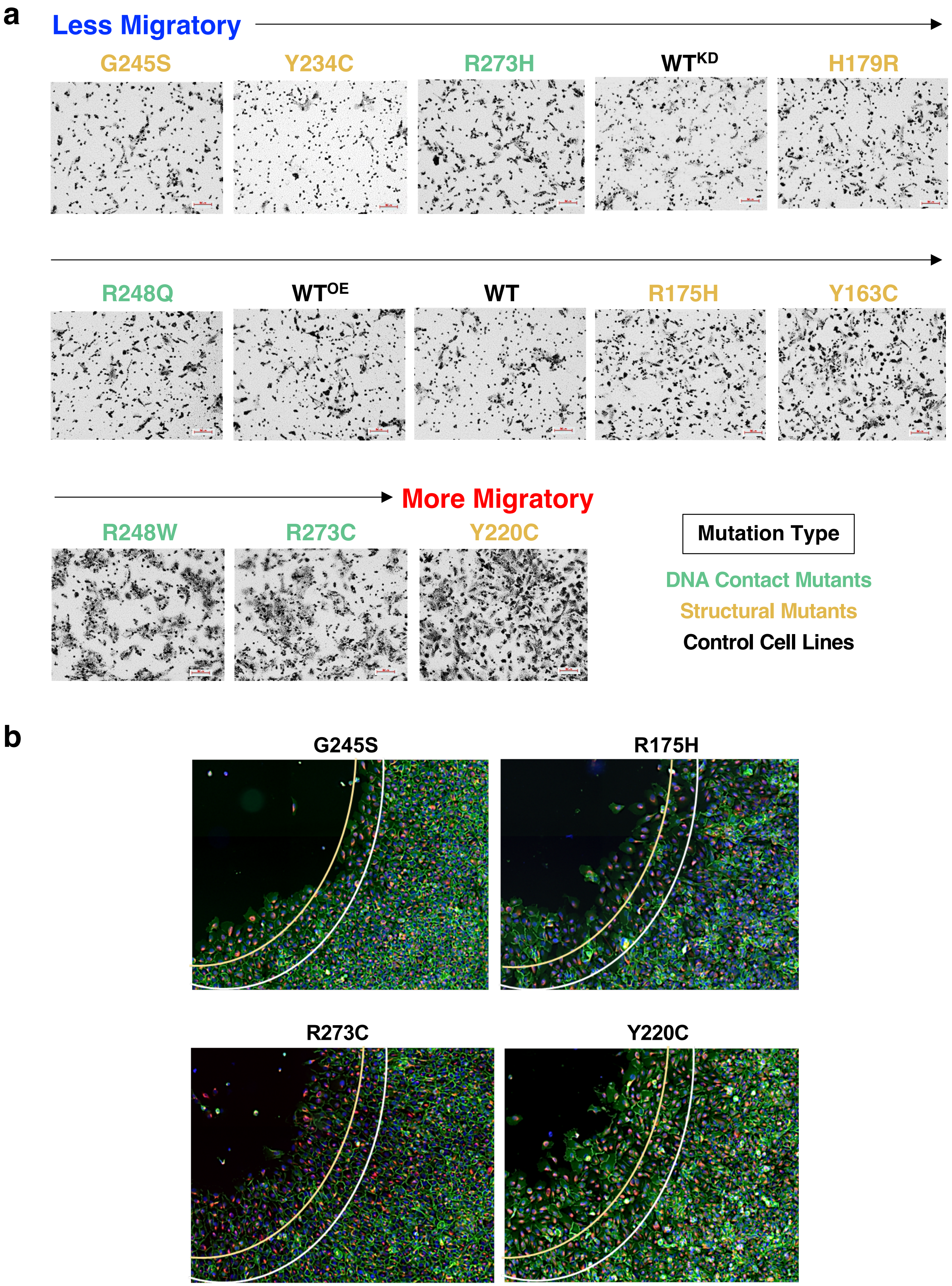

Supplementary Figure 4. Migration of MCF10A cell lines expressing different p53 mutants. a. Migratory potential of the MCF10A p53 mutants. Images of stained migratory cells under the transwell membrane are shown. The small black dots depict the pores in the membrane. Scale bar: 200μm. **b**. EMT markers- β-catenin and vimentin staining in the least (G245S), moderately (R175H) and most (R273C and Y220C) migratory p53 mutants. Cells were seeded in wells with a stopper. After the stopper was removed, the images were taken after 22 hrs. The white line shows the boundary of the stopper, and the yellow line the extent of migration of the least migratory mutant G245S. β-catenin was delocalized to the cytoplasm from the cell junctions in all the migratory cells.

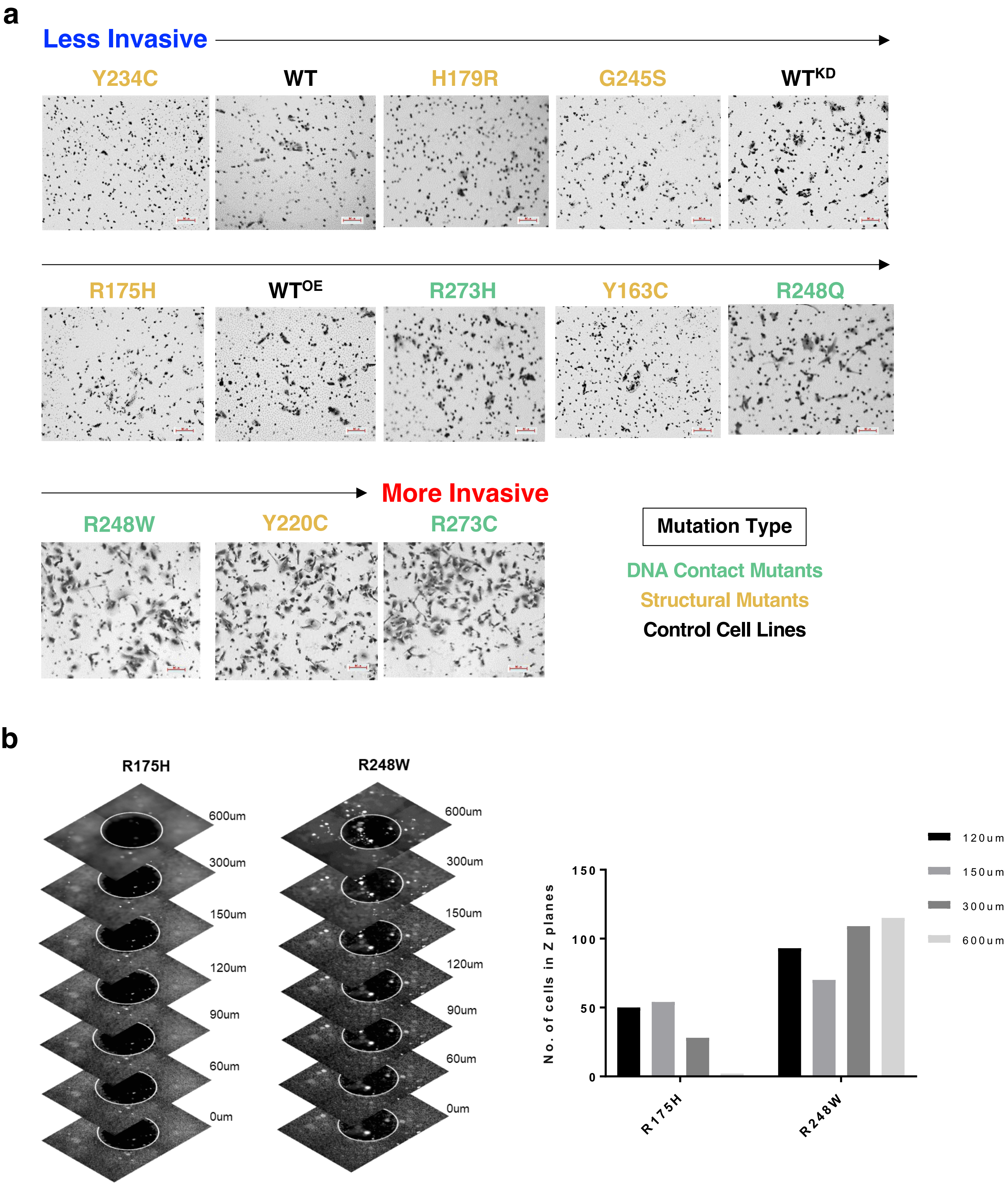

Supplementary Figure 5. Invasiveness of MCF10A cell lines expressing different p53 mutants. a. Invasiveness of the MCF10A p53 mutant-expressing cells. Images of stained invasive cells under the transwell membrane coated with Matrigel are shown. The small black dots depict the pores in the membrane. Scale bar: 200μm. **b**. Invasion of R175H (less invasive) and R248W (more invasive) mutants within 3D Matrigel matrix. Cross-sectional images at different Z focal planes are shown.

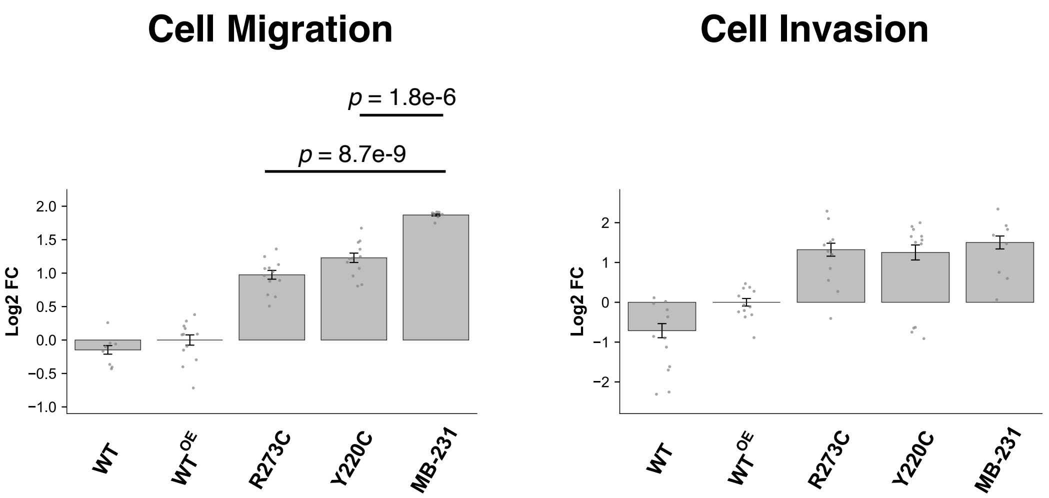

Supplementary Figure 6. Comparison of migration and invasion levels of MCF10A cell lines expressing different p53 mutants with MDA-MD231 breast cancer cells. Cell migration and invasion were measured for control cell lines (WT^{OE} and WT), 2 most invasive cell lines (R273C and Y220C), and the metastatic MDA-MD-231 TNBC cell line, and the values were normalized to the log2 fold changes over the WT^{OE} values. The *t*-test *p* values for significant differences are shown.

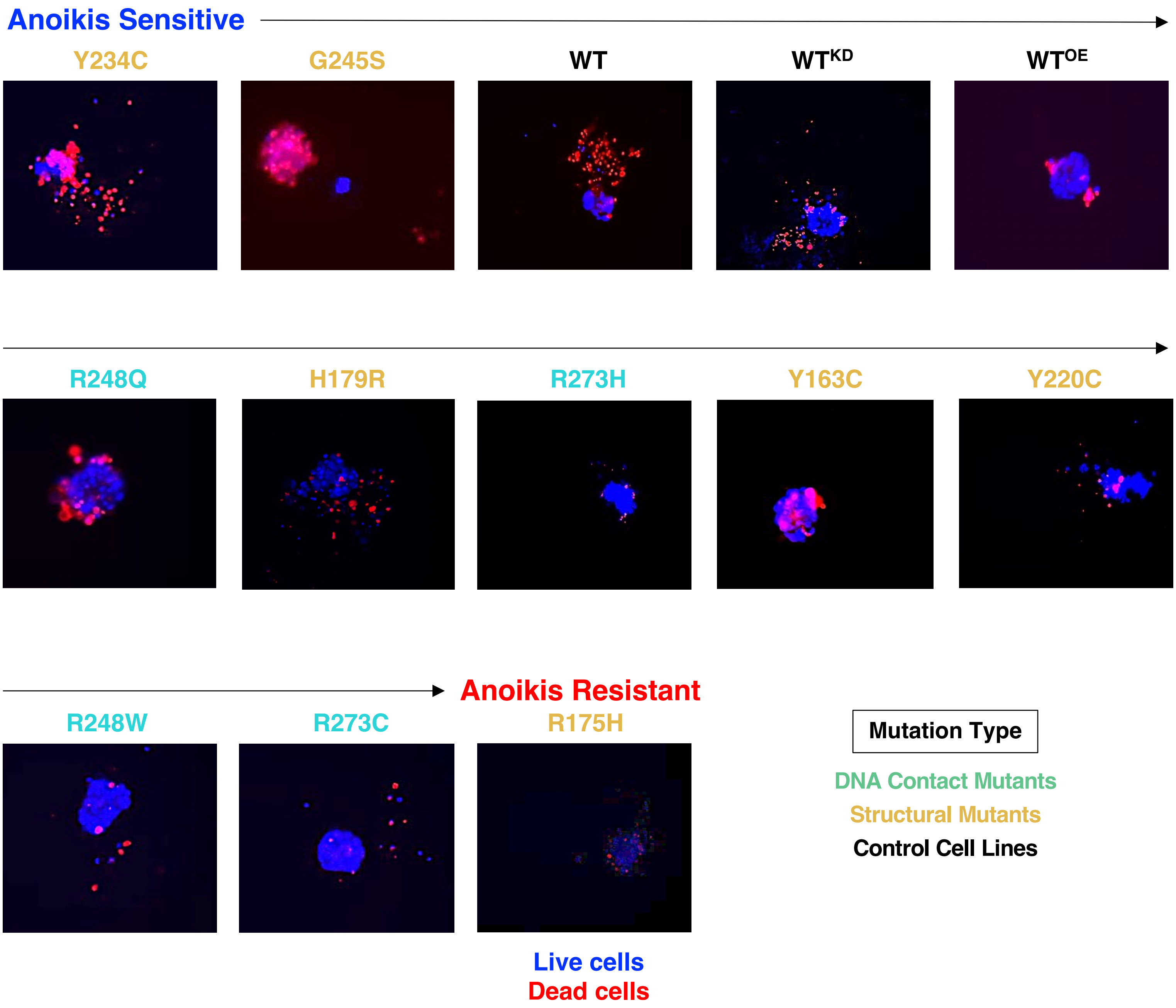

Supplementary Figure 7. Anoikis resistance of MCF10A cell lines expressing different p53 mutants. Cell aggregates were stained with ethidium bromide (dead cells, red) and Vybrant violet dye (live cells, blue). The total number of blue cells and red cells were counted to determine the death index (ratio of red or dead cells and blue or live cells). The p53 mutants are color-coded by mutant type as indicated.

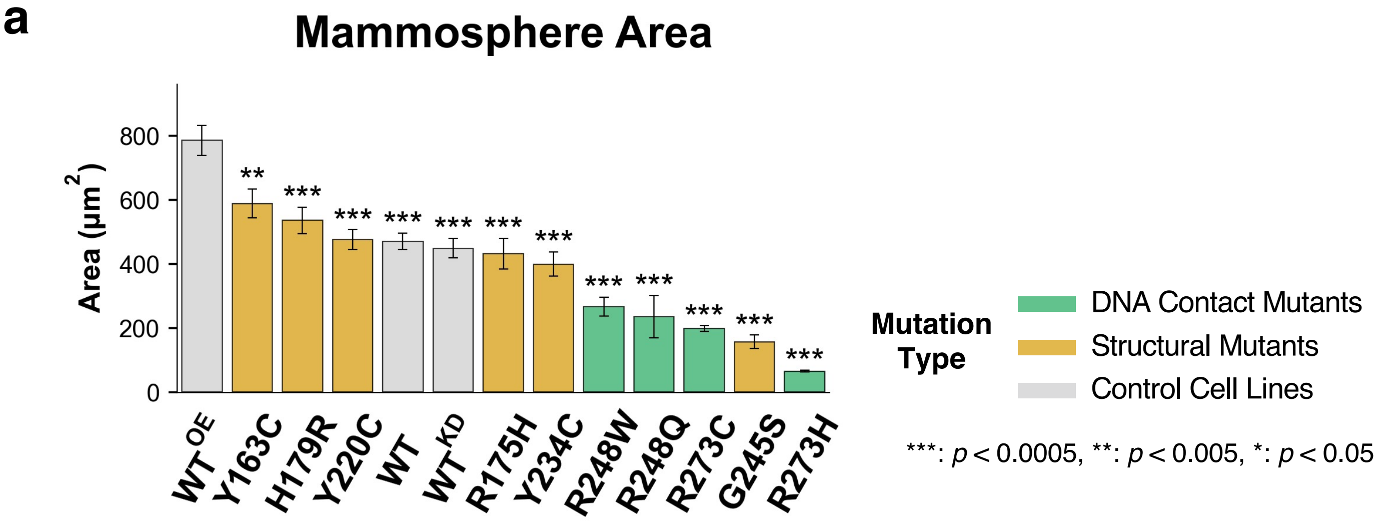

Mean	785.3	588.7	535.8	475.4	470.7	449.1	431.8	399.6	266.5	235.2	198.1	157.0	65.1
SEM	47.1	45.2	41.6	31.0	25.5	31.0	48.0	38.4	29.5	66.6	9.2	20.9	2.8
%CV	67.0	82.0	84.7	84.1	57.6	65.4	88.2	75.6	141.5	200.2	64.2	133.2	36.8
N	125	114	119	166	113	90	63	62	163	50	191	100	71

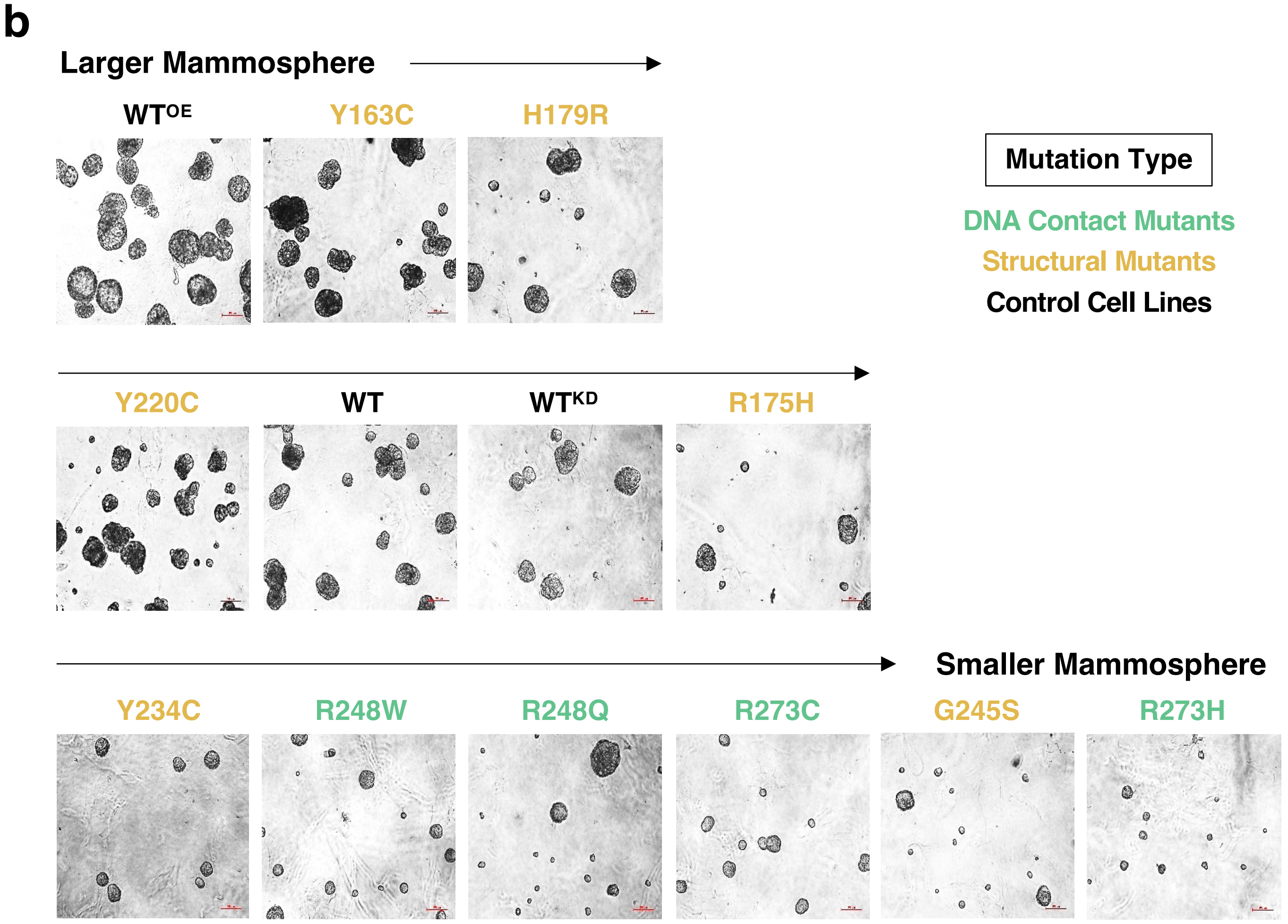

Supplementary Figure 8. Morphology of mammospheres derived from MCF10A cell lines expressing different p53 mutants. a. The mammosphere area (mm^2) were measured on day 9 in the on-top culture in 3D Matrigel bed. The mean, SD, SEM, %CV (coefficient of variation), and the number of mammosphere observed and analyzed are indicated in the table. **b**. A representative image for each cell line is shown. Scale bar: 100μm.

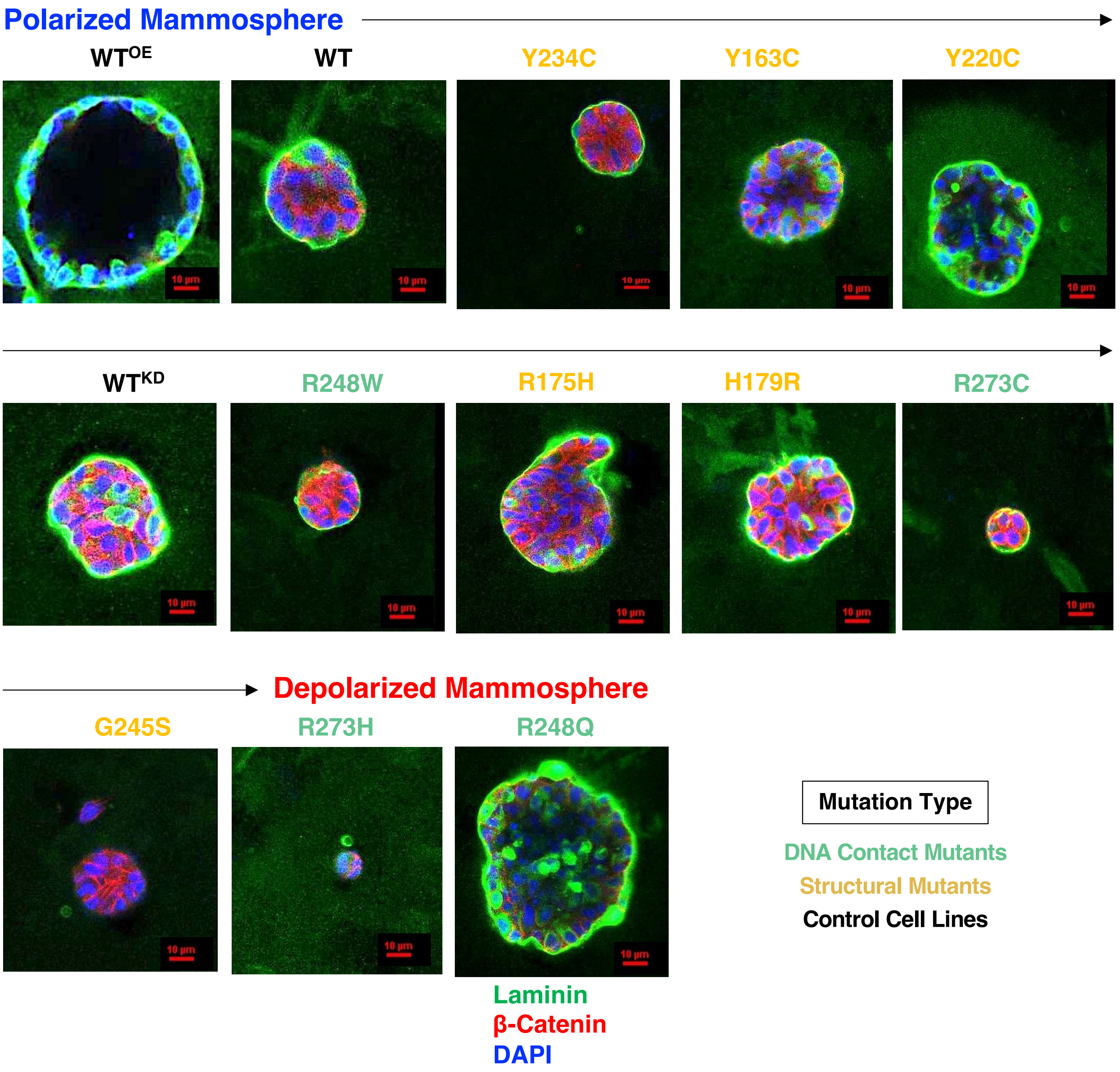

Supplementary Figure 9. Immunostaining of mammospheres derived from MCF10A cell lines expressing different p53 mutants. Mammospheres were immunostained for laminin (green), β-catenin (red), and the nucleus (DAPI, blue), and the representative confocal images of equatorial cross-section of mammospheres formed in 3D Matrigel matrix are shown. The cell lines were ordered by the degree of polarization shown in Figure 2b. Scale bar: 10μm.

a

TP53 Mutation	Number at Risk							5-Year Survival Rate
	Months							
	0	10	20	30	40	50	60	
H179R	3	2	2	1	1	1	1	100.0%
Y163C	3	3	2	2	2	2	2	100.0%
R248Q	15	14	13	13	12	11	10	79.4%
R175H	27	23	20	18	18	16	15	75.0%
Y220C	19	8	8	7	5	4	4	68.6%
WT	67	61	50	44	41	38	35	66.4%
R273H	15	15	9	9	6	6	6	66.5%
R248W	8	7	6	3	3	3	3	42.9%
R273C	6	6	6	4	2	2	2	33.3%

b

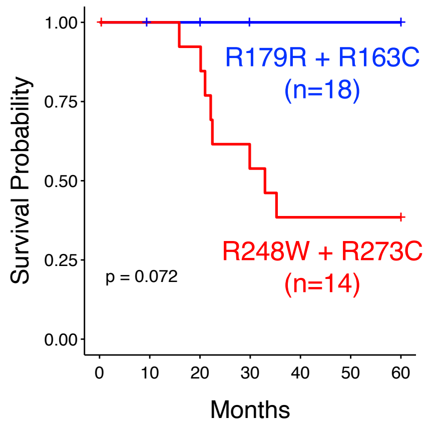

c

Cellular Phenotypes vs. 5-Year Survival Rates

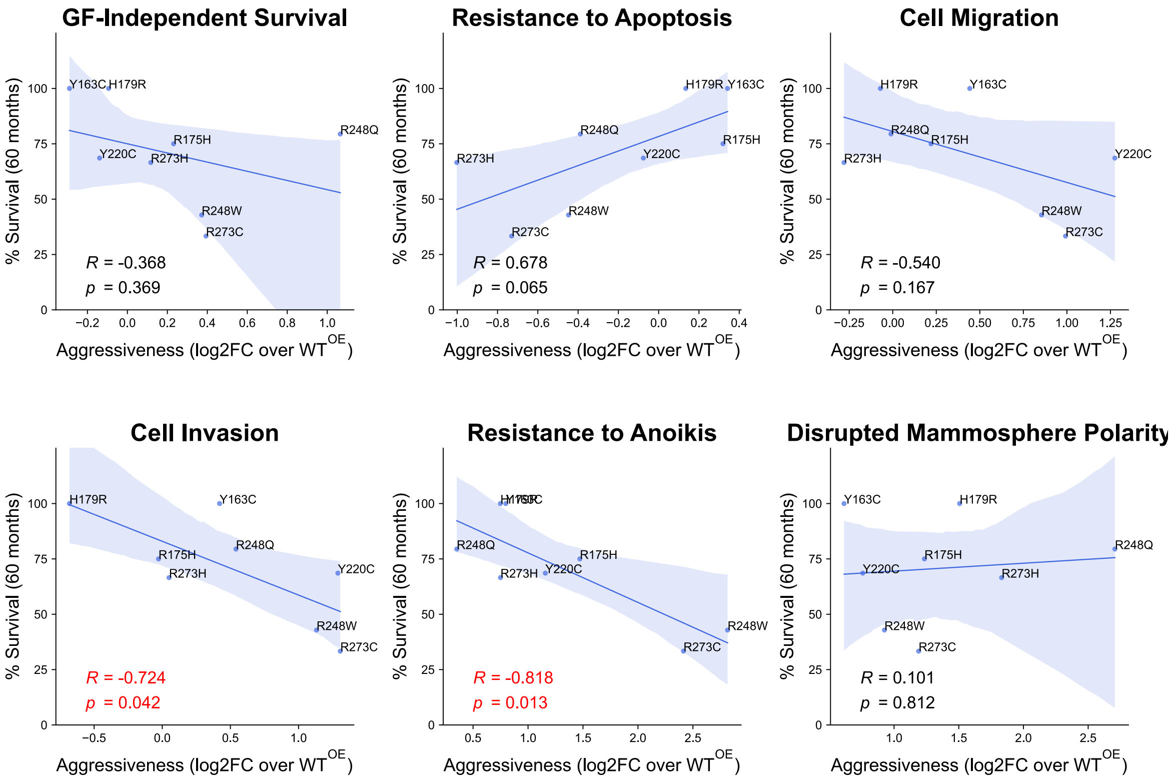

Supplementary Figure 10. Association of observed phenotypic aggressiveness with 5-year survival rates of basal-like breast cancer patients. a. Numbers at risk over 60 months and the survival rates for basal-like breast cancer patients with different missense are shown, and the Kaplan-Meier (KM) plot is in Figure 3b. **b**. A KM plot of between the patient groups with less (in blue) and more (in red) aggressive p53 mutants in cellular phenotypic assays. The log-rank test *p* value is shown. **c**. Regression plots between phenotype values and the 5-year survival rates are shown with the Pearson's *R* and the *p* values. The blue bands represent the 95% confidence intervals, and significant correlations were highlighted in red texts.

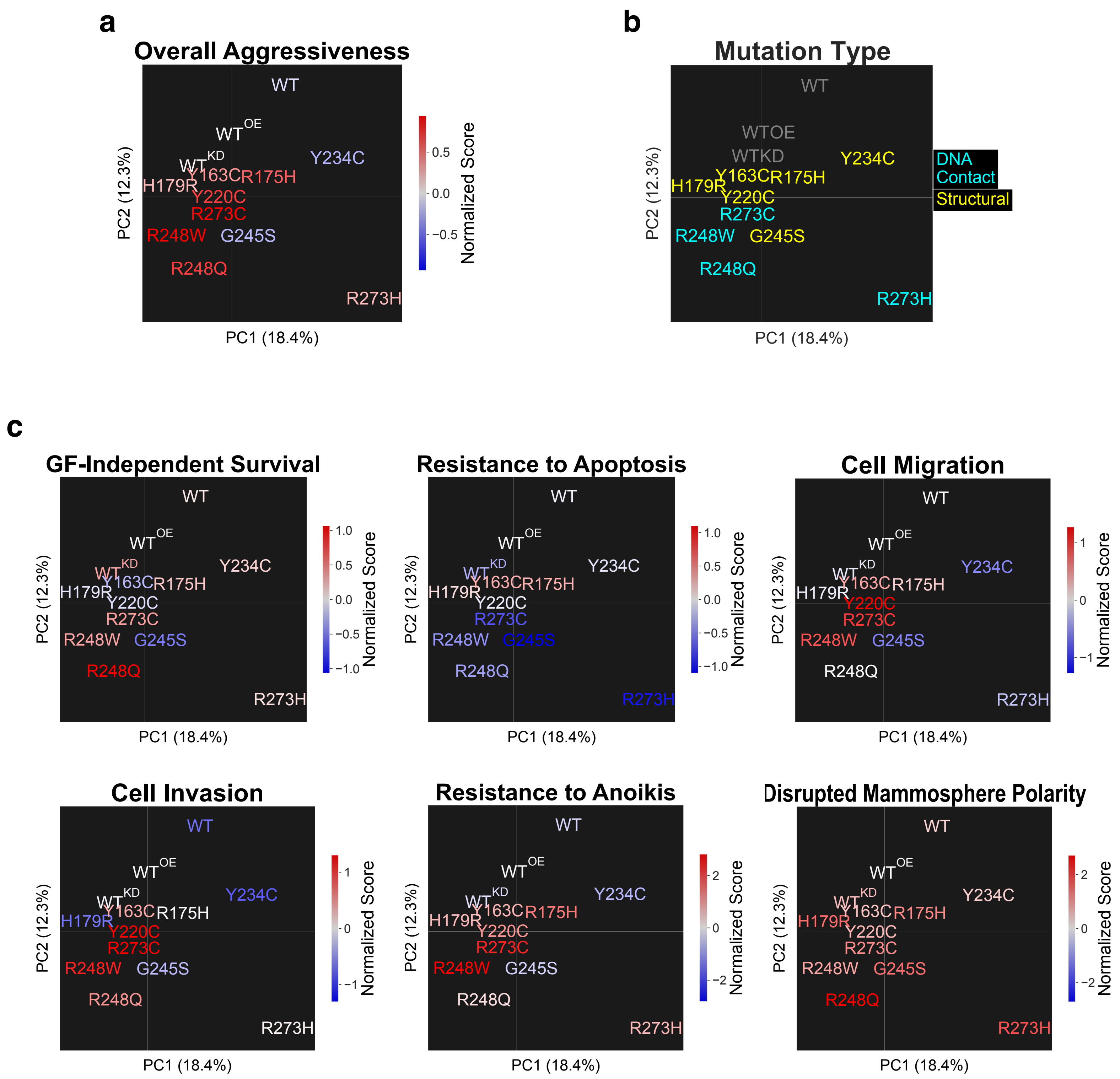

Supplementary Figure 11. PCA Analysis on RNA-Seq data of MCF10A cell lines expressing different p53 mutants. Variably expressed genes across all 13 cell lines (2,383 genes with coefficient of variation > 0.5 and mean TPM > 1) were used for PCA analysis. Cell line names were color-coded by overall aggressiveness (**a**), mutation type (**b**), and individual phenotypes (**c**) to visualize potential association with global gene expression profiles, as indicated.

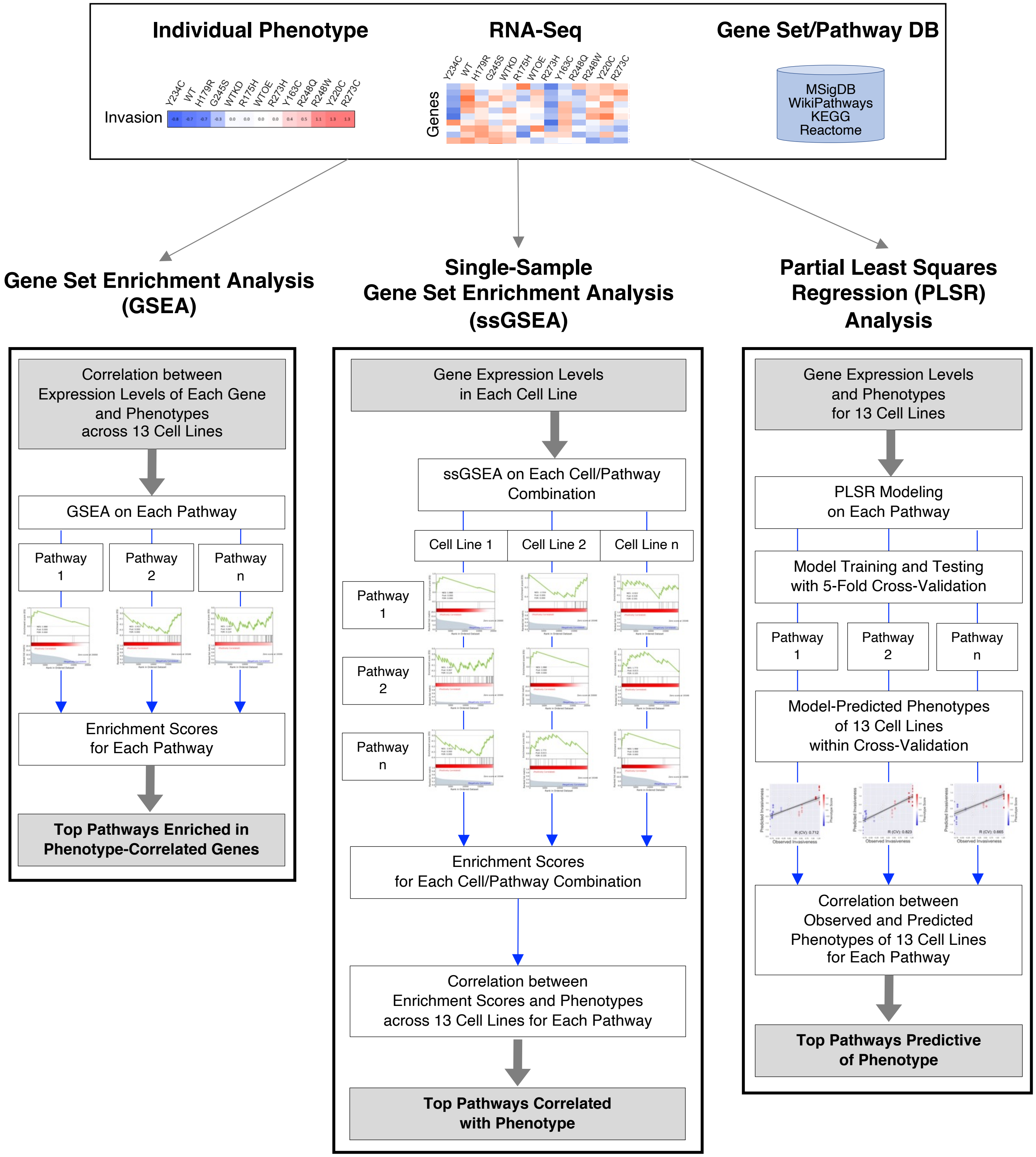

Supplementary Figure 12. Bioinformatics pipeline for identification of phenotype-associated gene sets and pathways based on RNA-Seq data. Schematic workflows of three different bioinformatics pipelines for pathway analysis of RNA-Seq data to assess the pathway-wise gene expression-to-phenotype association are shown. The GSEA pipeline examines the enrichment level of pathway genes in phenotype-correlated genes. The ssGSEA pipeline examines the correlation between the pathway enrichment scores of individual cell lines (obtained by ssGSEA) and the phenotypes. The PLSR pipeline measures expression-to-phenotype association of pathway genes by training machine-learning models to predict phenotypes based on expression values of pathway genes and testing the model performance by using cross-validation to minimize overfit.

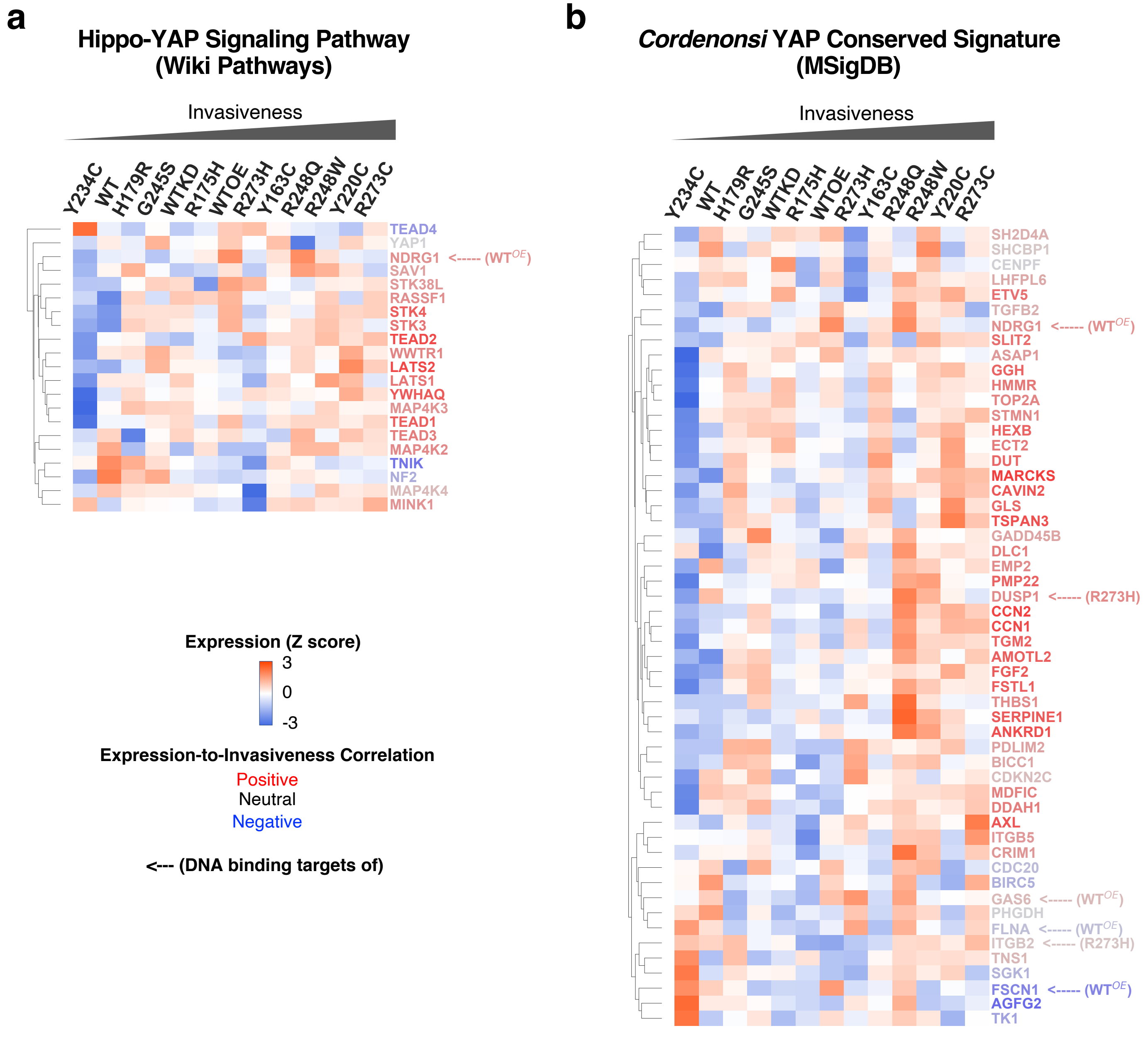

Supplementary Figure 13. Expression profiles of the invasion-associated Hippo/YAP/TAZ pathways in MCF10A cell lines expressing different p53 mutants. Expression levels in the 13-cell line panel of gene in the Hippo/YAP/TZA pathway (**a**, from Wiki Pathways) and the YAP/TAZ activation gene signature (**b**, from MSigDB Oncogenic Signatures) identified collectively from our pathway analysis are shown in heat maps. For visualization, the trend of expression across the cell lines, the expression values in RNA-Seq data were standardized to the Z scores for each gene. The font colors of the gene names represent the Pearson's R between expression values and invasiveness. Direct binding targets of either WT or mutant p53 proteins at the promoter region identified from ChIP-Seq analysis (Figure 6c) are annotated in the parentheses.

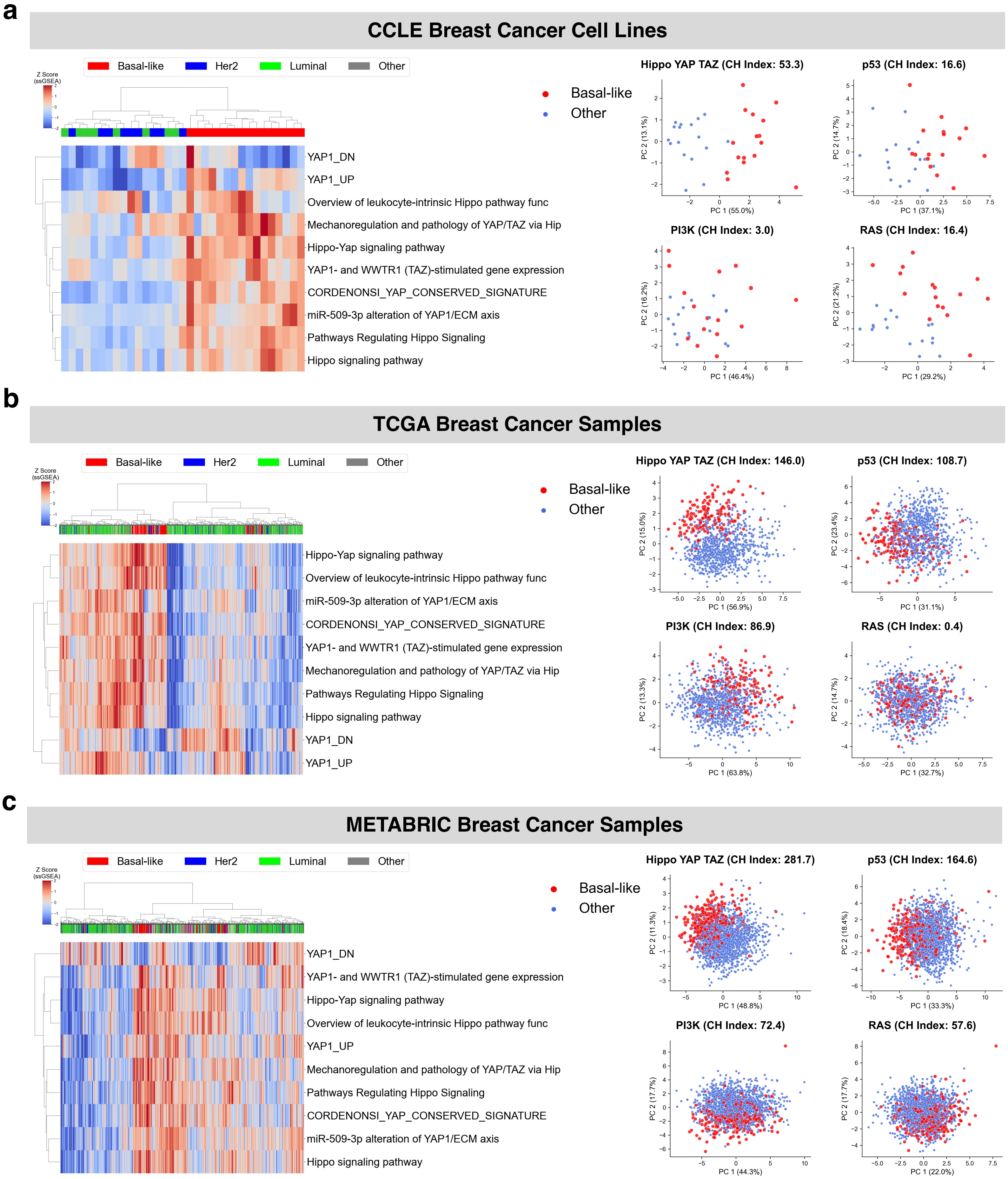

Supplementary Figure 14. Association of the Hippo pathway signature with basal-like breast cancer cells and tumors. ssGSEA scores on each cell line in CCLE (**a**) and sample in TCGA (**b**) and METABRIC (**c**) databases for the Hippo pathway terms were showed as clustergrams. (left panels). Molecular subtypes of samples are shown on the top. The datasets were subjected to PCA analysis, and the clustering strength of the basal-like samples was estimated by the the CH (Calinski-Harabasz) Indices.

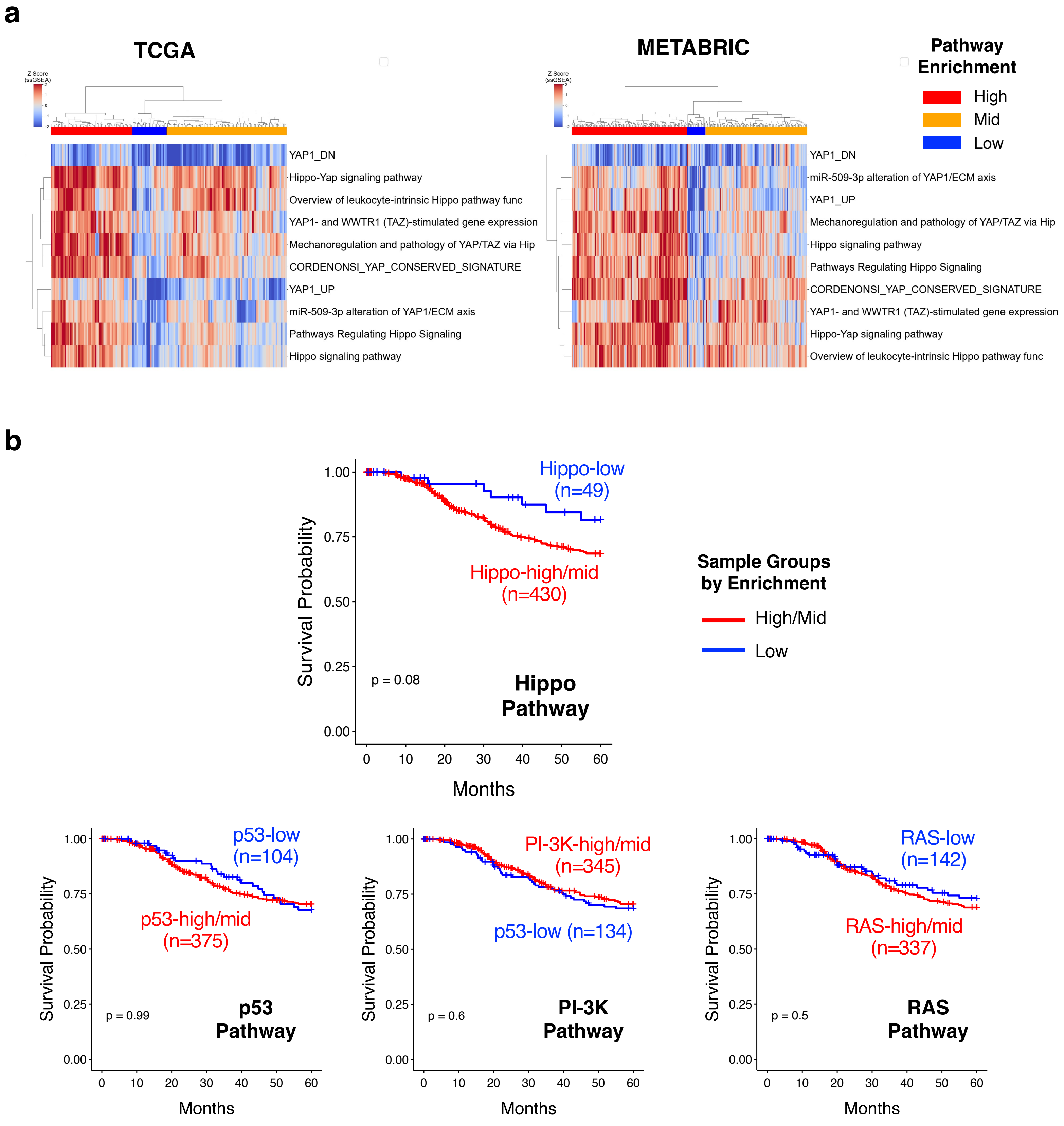

Supplementary Figure 15. Association of the Hippo pathway signature with basal-like breast cancer cells and tumors. **a**. The basal-like tumor samples in TCGA and METABRIC were clustered into 3 groups (high, mid, and low) by hierarchical clustering based on the mean of standardized ssGSEA enrichment scores for each pathway. **b**. The 5-year overall survival rates of the clusters with higher (*i.e.*, high and mid groups combined) and lower (low group) enrichments were examined. The log-rank test *p* values are shown.

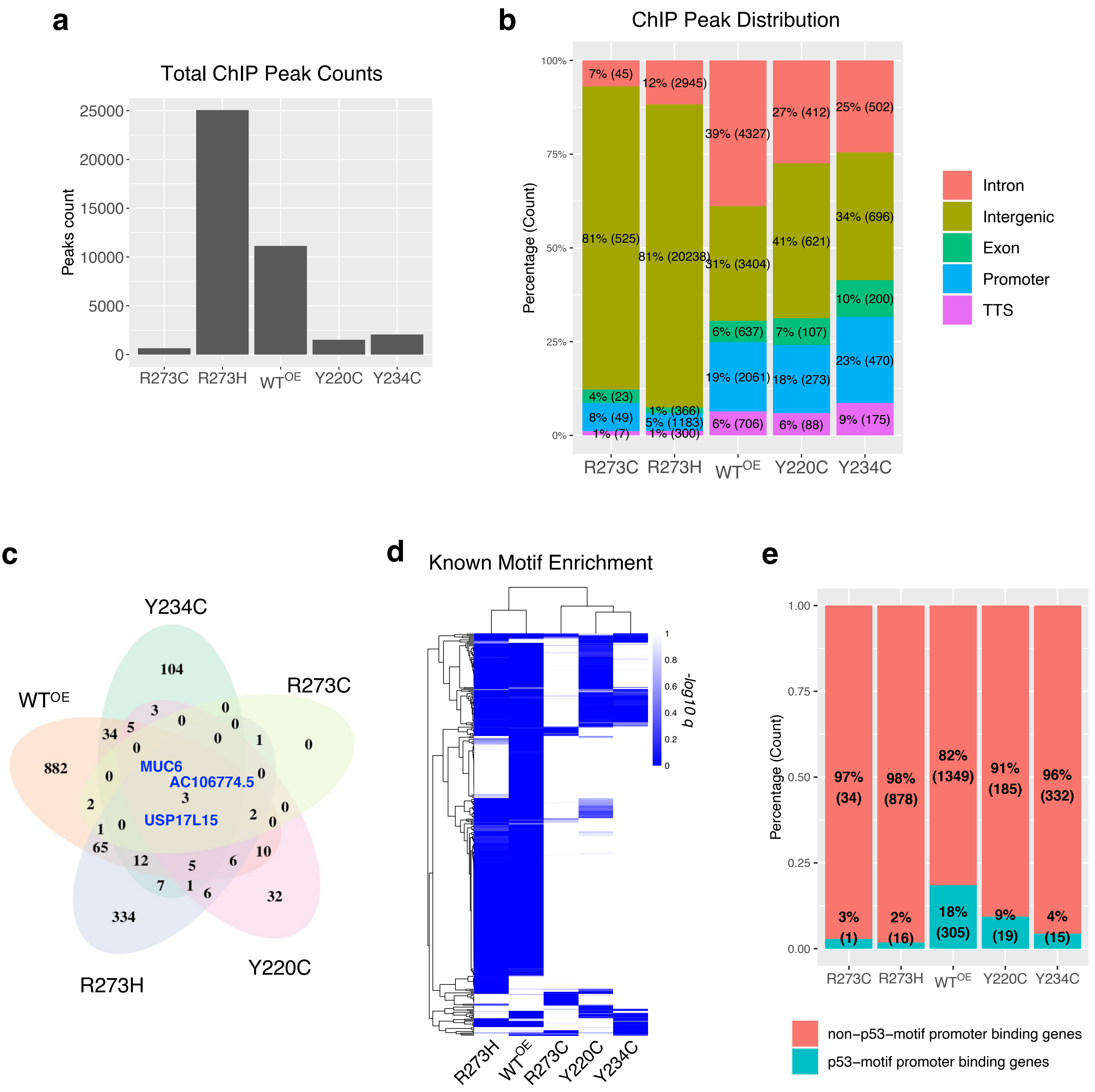

Supplementary Figure 16. ChIP-Seq analysis of selected MCF10A cell lines expressing different p53 mutants.
a. The total number of detected ChIP peaks (peak detection $q < 0.05$) in WT^{OE} and 4 mutant p53-expressing cell lines without treatment. **b**. Distribution of the detected ChIP peaks across different types of genomic regions are shown. **c**. Overlap of genes with peaks in the promoter region among different cell lines is shown in a Venn diagram. Three target genes common to all cell lines are shown in blue text. **d**. Heat map shows the enrichment profile (hypergeometric test q) of all known transcription factor binding motifs enriched among peaks (peak detection $q < 0.05$) located within the promoter regions. **e**. The fraction of genes with canonical p53 binding motif among all genes with peaks in the promoter region for each cell line is shown.

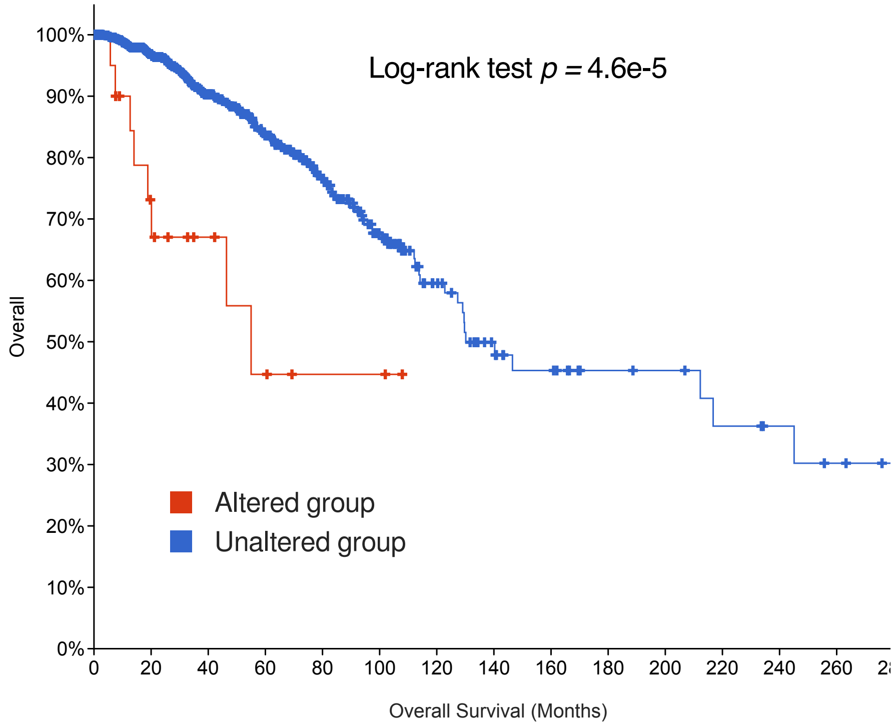

	Number of Cases, Total	Number of Cases, Deceased
Altered group	**21**	**8**
Unaltered group	**975**	**126**

Supplementary Figure 17. *CASZ1* gene mutation in breast cancer is associated with poor survival. Overall survival of patients with or without mutations in *CASZ1* was compared by the *cBioPortal* tool by using the TCGA PanCancer Atlas dataset.

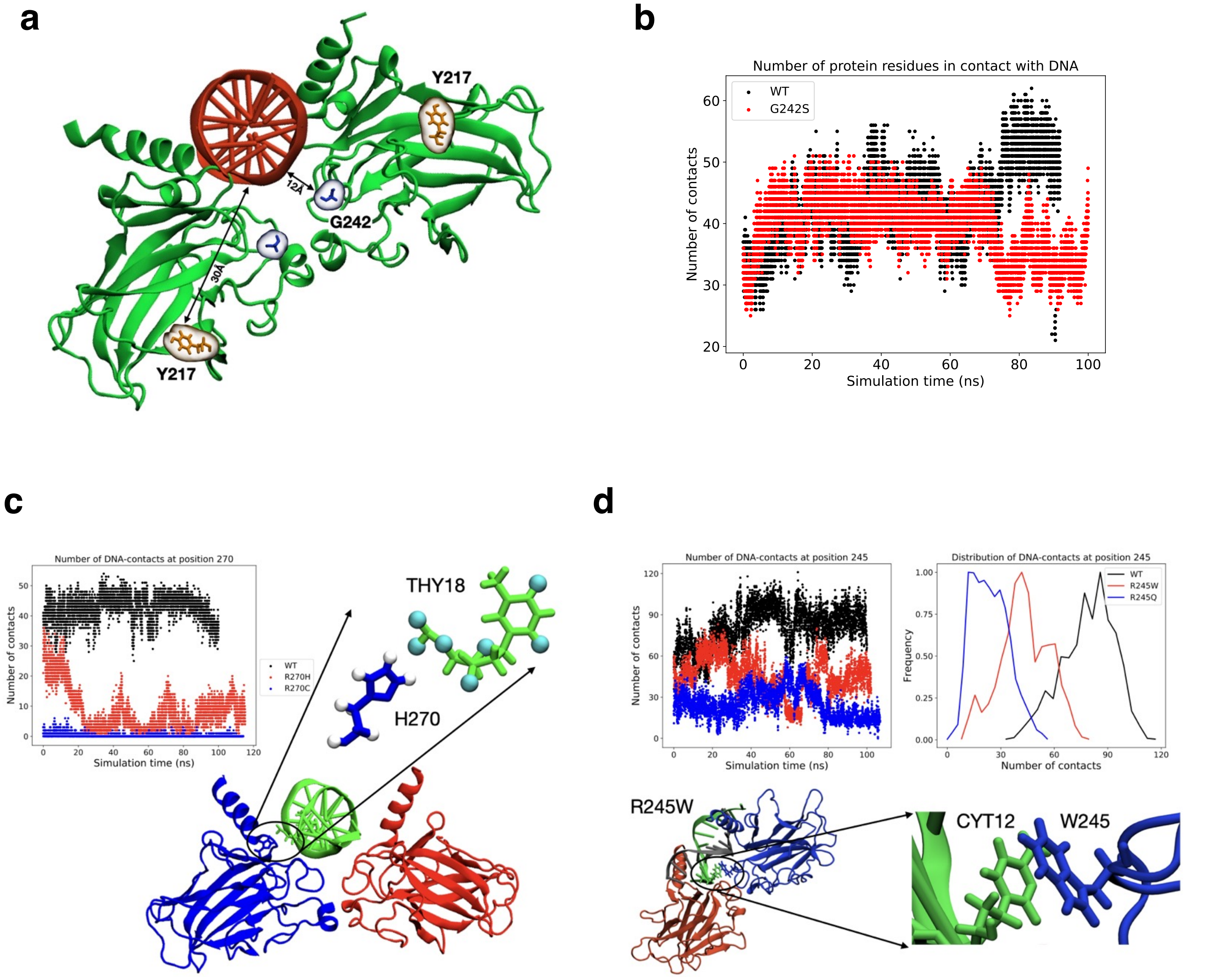

Supplementary Figure 18. Results from Molecular Dynamic (MD) simulation. The plots show protein-DNA interactions calculated over a 100 ns MD trajectory. The simulations have been performed with the mouse p53 mutant proteins (PDB ID: 3EXJ). The mouse p53 mutation positions 270, 242 and 245 correspond to 273, 245 and 248 in humans. **a**. Location of the mutation sites are highlighted, and the distance of Y217 and G242 from the DNA is shown. **b**. MD simulations show similar DNA binding dynamics for mutant G245S (red) and the WT protein (black), wherein the number of protein residues in contact with DNA is unchanged between WT and G245S. **c**. The plot shows the number of DNA atoms in contact with residue 273 in WT (black), R273H (red) and R273C (blue). The contact between DNA and protein decreases over time for p53 mutant R273H protein while mutant R273C does not make any contacts with the DNA. Histidine in R273H has weak electrostatic interactions with THY18 of DNA. **d**. Number of DNA atoms in contact with residue 248 in WT (black), R248W (red) and R248Q (blue). The DNA-protein contact for R248W mutant is weaker than R248Q, which is in turn weaker than WT p53 protein. Tryptophan in R248W has hydrophobic interactions with CYT12 of DNA.
