# Supplementary material for: Multidimensional quantitative phenotypic and molecular analysis reveals neomorphic behaviors of p53 missense mutants
Source: NPJ Breast Cancer. 2023 Sep 29;9:78. doi: 10.1038/s41523-023-00582-7 (PMC10541912; doi:10.1038/s41523-023-00582-7)
Supplement: Supplementary file 5 — nr-reporting-summary [file 41523_2023_582_MOESM5_ESM.pdf]

nature portfolio

Corresponding author(s): Josh LaBaer and Jin Park

Last updated by author(s): 08/28/2023

Reporting Summary

Nature Portfolio wishes to improve the reproducibility of the work that we publish. This form provides structure for consistency and transparency in reporting. For further information on Nature Portfolio policies, see our Editorial Policies and the Editorial Policy Checklist.

Statistics

For all statistical analyses, confirm that the following items are present in the figure legend, table legend, main text, or Methods section.

n/a	Confirmed	
☐	☒	The exact sample size (*n*) for each experimental group/condition, given as a discrete number and unit of measurement
☐	☒	A statement on whether measurements were taken from distinct samples or whether the same sample was measured repeatedly
☐	☒	The statistical test(s) used AND whether they are one- or two-sided<br>*Only common tests should be described solely by name; describe more complex techniques in the Methods section.*
☐	☒	A description of all covariates tested
☐	☒	A description of any assumptions or corrections, such as tests of normality and adjustment for multiple comparisons
☐	☒	A full description of the statistical parameters including central tendency (e.g. means) or other basic estimates (e.g. regression coefficient) AND variation (e.g. standard deviation) or associated estimates of uncertainty (e.g. confidence intervals)
☐	☒	For null hypothesis testing, the test statistic (e.g. *F*, *t*, *r*) with confidence intervals, effect sizes, degrees of freedom and *P* value noted<br>*Give P values as exact values whenever suitable.*
☒	☐	For Bayesian analysis, information on the choice of priors and Markov chain Monte Carlo settings
☒	☐	For hierarchical and complex designs, identification of the appropriate level for tests and full reporting of outcomes
☒	☐	Estimates of effect sizes (e.g. Cohen's *d*, Pearson's *r*), indicating how they were calculated

Our web collection on statistics for biologists contains articles on many of the points above.

Software and code

Policy information about availability of computer code

Data collection	RNA-Seq data on cell lines were downloaded from CCLE. RNA-Seq data on breast cancer samples in TCGA (BRCA PanCancer Atlas version) and METABRIC (Nature 2012 & Nat Commun 2016 version) were downloaded from cBIO Portal. Pathway gene sets were downloaded from MSigDB v7.2.
Data analysis	RNA-Seq data were processed by STAR v020201. ChIP-Seq data were analyzed by Bowtie2 v2.1.0, Samtools v1.7, PICARD v2.18.3, MACS2 v2.1.2.20181017, Homer v4.9.1, and MEME Suite v5.0.1. PLSR analysis was done by pyChemometrics V0.13.3 and scikit-learn v0.23.2. Molecular dynamics simulation was done by Visual Molecular Dynamics (VMD) and NAMD v2.13. More detailed information on all software and the run parameters are listed in the Methods and the Supplementary Methods.

For manuscripts utilizing custom algorithms or software that are central to the research but not yet described in published literature, software must be made available to editors and reviewers. We strongly encourage code deposition in a community repository (e.g. GitHub). See the Nature Portfolio guidelines for submitting code & software for further information.

Data

Policy information about availability of data

All manuscripts must include a data availability statement. This statement should provide the following information, where applicable:

- Accession codes, unique identifiers, or web links for publicly available datasets
- A description of any restrictions on data availability
- For clinical datasets or third party data, please ensure that the statement adheres to our policy

Raw sequencing data for RNA-Seq and ChIP-Seq experiments were deposited to the Gene Expression Omnibus (GEO) with an accession number of GSE162341 (https://www.ncbi.nlm.nih.gov/geo/query/acc.cgi?acc= GSE162341), which is accessible to reviewers (reviewer's token: mvwxyiwszhmjpij). Phenotype data, processed RNA-Seq/ChIP-Seq data, and pathway analysis results used for figures are provided in Supplementary Tables.

Research involving human participants, their data, or biological material

Policy information about studies with human participants or human data. See also policy information about sex, gender (identity/presentation), and sexual orientation and race, ethnicity and racism.

Reporting on sex and gender	*Use the terms sex (biological attribute) and gender (shaped by social and cultural circumstances) carefully in order to avoid confusing both terms. Indicate if findings apply to only one sex or gender; describe whether sex and gender were considered in study design; whether sex and/or gender was determined based on self-reporting or assigned and methods used. Provide in the source data disaggregated sex and gender data, where this information has been collected, and if consent has been obtained for sharing of individual-level data; provide overall numbers in this Reporting Summary. Please state if this information has not been collected. Report sex- and gender-based analyses where performed, justify reasons for lack of sex- and gender-based analysis.*
Reporting on race, ethnicity, or other socially relevant groupings	*Please specify the socially constructed or socially relevant categorization variable(s) used in your manuscript and explain why they were used. Please note that such variables should not be used as proxies for other socially constructed/relevant variables (for example, race or ethnicity should not be used as a proxy for socioeconomic status). Provide clear definitions of the relevant terms used, how they were provided (by the participants/respondents, the researchers, or third parties), and the method(s) used to classify people into the different categories (e.g. self-report, census or administrative data, social media data, etc.) Please provide details about how you controlled for confounding variables in your analyses.*
Population characteristics	*Describe the covariate-relevant population characteristics of the human research participants (e.g. age, genotypic information, past and current diagnosis and treatment categories). If you filled out the behavioural & social sciences study design questions and have nothing to add here, write "See above."*
Recruitment	*Describe how participants were recruited. Outline any potential self-selection bias or other biases that may be present and how these are likely to impact results.*
Ethics oversight	*Identify the organization(s) that approved the study protocol.*

Note that full information on the approval of the study protocol must also be provided in the manuscript.

Field-specific reporting

Please select the one below that is the best fit for your research. If you are not sure, read the appropriate sections before making your selection.

☒ Life sciences ☐ Behavioural & social sciences ☐ Ecological, evolutionary & environmental sciences

For a reference copy of the document with all sections, see nature.com/documents/nr-reporting-summary-flat.pdf

Life sciences study design

All studies must disclose on these points even when the disclosure is negative.

Sample size	To detect log2 fold change of +/-1.0 from the reference WTOE cells with SD of 0.5 (assumed from our previous observations) at the significance level of 0.05, six samples per group would provide 94% power. Actual samples sizes per group used in this study ranged from 6 to 191 (average N = 35).
Data exclusions	The data from samples that showed excessive cell death or with values out of measurable range were excluded.
Replication	All assays were done on two to six batches of independent batches of cell lines in triplicates, and all results were merged for data analyses.
Randomization	Not applicable
Blinding	Not applicable

Reporting for specific materials, systems and methods

We require information from authors about some types of materials, experimental systems and methods used in many studies. Here, indicate whether each material, system or method listed is relevant to your study. If you are not sure if a list item applies to your research, read the appropriate section before selecting a response.

Materials & experimental systems

n/a	Involved in the study
☐	☒ Antibodies
☐	☒ Eukaryotic cell lines
☒	☐ Palaeontology and archaeology
☒	☐ Animals and other organisms
☒	☐ Clinical data
☒	☐ Dual use research of concern
☒	☐ Plants

Methods

n/a	Involved in the study
☐	☒ ChIP-seq
☒	☐ Flow cytometry
☒	☐ MRI-based neuroimaging

Antibodies

Antibodies used	p53 antibody (Sigma, Cat # P6874 Lot # 013M4847V), V5 tag antibody (Cell Signaling Technologies, Cat # 13202S Lot # 4), β-Catenin antibody (Cell Signaling Technologies, Cat # 2677S Lot # 1), Laminin antibody (Millipore, Cat # MAB19562, Lot year 2014), Vimentin antibody (Cell Signaling Technology, Cat # 5741 Lot # 1)
Validation	All antibodies used in this study were from commercial sources. The manufacturer websites provide validation data.

Eukaryotic cell lines

Policy information about cell lines and Sex and Gender in Research

Cell line source(s)	MCF-10A cell line was purchased from ATCC (Cat # CRL-10317).
Authentication	MCF-10A cells were purchased from in 2010 ATCC with the certificate of analysis.
Mycoplasma contamination	The parental MCF-10A and all p53 mutant cell lines were tested and confirmed negative for mycoplasma contamination, which was done by PCR.
Commonly misidentified lines (See ICLAC register)	*Name any commonly misidentified cell lines used in the study and provide a rationale for their use.*

Plants

Seed stocks	*Report on the source of all seed stocks or other plant material used. If applicable, state the seed stock centre and catalogue number. If plant specimens were collected from the field, describe the collection location, date and sampling procedures.*
Novel plant genotypes	*Describe the methods by which all novel plant genotypes were produced. This includes those generated by transgenic approaches, gene editing, chemical/radiation-based mutagenesis and hybridization. For transgenic lines, describe the transformation method, the number of independent lines analyzed and the generation upon which experiments were performed. For gene-edited lines, describe the editor used, the endogenous sequence targeted for editing, the targeting guide RNA sequence (if applicable) and how the editor was applied.*
Authentication	*Describe any authentication procedures for each seed stock used or novel genotype generated. Describe any experiments used to assess the effect of a mutation and, where applicable, how potential secondary effects (e.g. second site T-DNA insertions, mosiacism, off-target gene editing) were examined.*

ChIP-seq

Data deposition

☒ Confirm that both raw and final processed data have been deposited in a public database such as GEO.

☒ Confirm that you have deposited or provided access to graph files (e.g. BED files) for the called peaks.

Data access links *May remain private before publication.*	GSE162341 (https://www.ncbi.nlm.nih.gov/geo/query/acc.cgi?acc= GSE162341)
Files in database submission	FASTQ files for RNA-Seq and ChIP-Seq. NarrowPeak (BED format) files for ChIP-Seq.

Genome browser session
(e.g. UCSC)

Provide a link to an anonymized genome browser session for "Initial submission" and "Revised version" documents only, to enable peer review. Write "no longer applicable" for "Final submission" documents.

Methodology

Replicates

ChIP-seq was performed on a single sample for each p53 mutant expressing cell line.

Sequencing depth

For the R273C mutant, the IP has a total of 86070616 reads, with 69140728 reads being uniquely mapped. The Input has a total of 50553660 reads, out of which 40203272 reads are uniquely mapped. For the R273H mutant, the IP has a total of 118909370 reads, with 96402864 reads being uniquely mapped. The Input has a total of 47754944 reads, out of which 37416690 reads are uniquely mapped. For the WT-OE, the IP has a total of 111881528 reads, with 82792119 reads being uniquely mapped. The Input has a total of 49997780 reads, out of which 39678326 reads are uniquely mapped. For the Y220C mutant, the IP has a total of 90848278 reads, with 71622926 reads being uniquely mapped. The Input has a total of 44682360 reads, out of which 34524459 reads are uniquely mapped. For the Y234C mutant, the IP has a total of 94601872 reads, with 71491403 reads being uniquely mapped. The Input has a total of 42069912 reads, out of which 32696372 reads are uniquely mapped. Paired-end sequencing was used in all experiments, and most of the read lengths are 76bp.

Antibodies

Anti-V5 tag antibody was purchased from the Cell Signaling Technology (cat #13202S, lot #4).

Peak calling parameters

macs2 callpeak -t cell_1_IP_sorted.bam -c cell_1_Input_sorted.bam -B -g hs -s 300 --call-summits -q 0.05 -n cell_1 -f BAMPE

Data quality

In the peak calling step for R273C, a total of 7 peaks were identified with an FDR of less than 0.05 and a fold-enrichment greater than 5. In the peak calling step for R273H, a total of 8581 peaks were identified with an FDR of less than 0.05 and a fold-enrichment greater than 5. In the peak calling step for WT-OE, a total of 41 peaks were identified with an FDR of less than 0.05 and a fold-enrichment greater than 5. In the peak calling step for Y220C, a total of 33 peaks were identified with an FDR of less than 0.05 and a fold-enrichment greater than 5. In the peak calling step for Y234C, a total of 83 peaks were identified with an FDR of less than 0.05 and a fold-enrichment greater than 5.

Software

Quality of the reads were analyzed using FastQC (v0.10.1). Paired-ended reads were mapped to the reference human genome (GRCh38.p92/hg38) end to end using Bowtie2 (v2.1.0). Non-primary alignment, unmapped reads were removed by Samtools (v1.7). Duplication rates were calculated by PICARD (v2.18.3) (duplication rate ranges from 18% to 88%). MACS2 (v2.1.2.20181017) was used for peak calling using BAMPE mode with the q-value cutoff of 0.05 by using the input genomic DNA sequencing data as the background. Peaks were annotated using Homer toolkit (Homer v4.9.1) 9, which was also been utilized for motif finding. Regions located between -100 and 2,500 bp upstream of the nearest transcription start sites (TSS) were defined as the promoter region. Known and de novo motifs were called by Homer toolkit. De novo motifs comparison was done by Tomtom in MEME Suite (v5.0.1). More details can be found in the Supplemental Methods.
